# *Escherichia-Shigella* expansion and metabolite dysregulation in type 3c diabetes: linking microbiome alterations to exocrine pancreatic insufficiency

**DOI:** 10.3389/fendo.2026.1786756

**Published:** 2026-03-27

**Authors:** Erika Kvalem Soto, Valeria Wagner, Julia Engl, Michael Mederer, Veronika Cibulkova, Johanna Piater, Benedikt Schäfer, Elena Dunzendorfer, Silvio Waschina, Susanne Kaser, Konrad Aden, Zlatko Trajanoski, Herbert Tilg, Maria Effenberger

**Affiliations:** 1 Biocenter, Institute of Bioinformatics, Medical University of Innsbruck, Innsbruck, Austria; 2 Department of Internal Medicine I, Gastroenterology, Hepatology, Endocrinology & Metabolism, Medical University of Innsbruck, Innsbruck, Austria; 3 Department of Medicine, Hospital of Brixen (SABES-ASDAA), Teaching Hospital of Paracelsus Medical University, Bessanone-Brixen, Italy; 4 Institute for Human Nutrition and Food Science, Division Nutriinformatics, Christian-Albrechts-University of Kiel, Kiel, Germany; 5 Institute of Clinical Molecular Biology, Christian-Albrechts-University and University Hospital Schleswig-Holstein, Kiel, Germany; 6 Department of Internal Medicine I., Christian-Albrechts-University and University Hospital Schleswig-Holstein, Kiel, Germany

**Keywords:** diabetes, EPI, gut microbiome, metabolome, T3cDM

## Abstract

Emerging evidence supports a bidirectional gut–pancreas axis in which microbial dysbiosis, barrier dysfunction, and altered metabolite fluxes contribute to pancreatogenic diabetes (T3cDM). Whether gut microbial changes reflect systemic metabolic disturbances or primarily arise from exocrine pancreatic insufficiency (EPI) remains unclear. We profiled the gut microbiome of 48 outpatients with T3cDM, type 1 diabetes (T1DM), and healthy controls. Genus-level 16S rRNA data were analyzed using cross-validated LASSO logistic regression and patient-specific community metabolic models. T3cDM showed reduced α-diversity and distinct β-diversity compared with T1DM and controls. Key compositional shifts included enrichment of *Enterobacteriaceae* (notably *Escherichia–Shigella*) and *Streptococcaceae* in T3cDM. LASSO models discriminated T3cDM from T1DM (AUC 0.867; accuracy 0.818), highlighting *Blautia, Escherichia–Shigella, Streptococcus, Clostridium, and Faecalibacterium* as predictors. Metabolic modelling indicated elevated *Escherichia–Shigella* growth in T3cDM and disease-specific metabolite fluxes. Gut microbial shifts in T3cDM predominantly reflect EPI rather than systemic metabolic disturbances characteristic of T1DM, underscoring the central role of exocrine pancreatic dysfunction in shaping the gut microbiome and its metabolic activity.

## 1 Introduction

Pancreatogenic diabetes mellitus (T3cDM) is defined as diabetes secondary to exocrine pancreatic disease, including total or partial pancreatectomy, pancreatic agenesis, acute and chronic pancreatitis, cystic fibrosis, haemochromatosis, and pancreatic ductal adenocarcinoma (1). Despite representing only, a minor fraction of pancreatic volume, islet cell loss or dysfunction frequently leads to impaired glucose homeostasis in these conditions. Although grouped under a single entity, the pathophysiology varies by etiology and has been best characterized in chronic pancreatitis, pancreatic cancer, and cystic fibrosis (2–4).

Accumulating data suggest that gut-derived microbes modulate pancreatic disease progression through both local and systemic immune responses (5–7). Microbial components can activate pattern recognition receptors on pancreatic epithelial and immune cells, triggering the release of pro-inflammatory cytokines and chemokines that contribute to tissue injury. They also influence the production of antimicrobial peptides, alter the composition and activity of intrapancreatic immune cell populations, and modulate systemic inflammatory pathways, amplifying pancreatic damage. Notably, the pancreas does not harbor a resident microbiota under normal conditions; thus, dysbiosis in the gut may act as a primary driver of pancreatic inflammation and exocrine pancreatic insufficiency (EPI) (6, 8). These findings underscore the critical role of the gut–pancreas axis in modulating both local injury and broader disease manifestations.

Growing evidence indicates that pancreatic digestive enzymes are integral to systemic metabolic homeostasis by shaping nutrient sensing along the gut–pancreas axis. Lipolytic and proteolytic products generated intraluminally engage enteroendocrine sensors on K- and L-cells—long-chain fatty acids activate FFAR4/GPR120 to promote incretin secretion, while amino acids and peptones signal via receptors such as the calcium-sensing receptor (CaSR)—thereby modulating incretin release and downstream insulinotropic responses (9–12). Pancreatic enzyme activity also shapes bile acid flux and composition in the intestinal lumen, influencing farnesoid X receptor (FXR) and Takeda G-protein receptor 5 (TGR5) signaling pathways that regulate GLP-1 secretion, energy expenditure, and glucose homeostasis (11, 13). Bile acids can stimulate GLP-1 release from intestinal L-cells through TGR5 signaling and interact with FXR to modulate metabolic pathways relevant to glycemic control (14). In parallel, the extent and site of macronutrient hydrolysis determine substrate delivery to the colon and thereby microbial fermentation and short-chain fatty acid (SCFA) production; SCFAs subsequently act through FFAR2 and FFAR3 to influence incretin secretion, intestinal barrier function, and host energy balance (12, 15). Together, these mechanisms underscore that exocrine pancreatic function is inseparable from metabolic regulation, linking digestive capacity to enteroendocrine signaling, bile acid–host crosstalk, and microbiota-derived metabolites that collectively shape glycemic and energy homeostasis (11, 12).

The metabolome has emerged as a key mediator linking pancreatic dysfunction to systemic metabolic consequences. In T3cDM, alterations in lipid, amino acid, and bile acid metabolism reflect both endocrine impairment and EPI (16, 17). Distinct metabolomic signatures have been reported in acute and chronic pancreatitis, with shifts in energy substrates and oxidative stress pathways paralleling disease severity (18, 19). Disrupted amino acid and branched-chain fatty acid profiles in T3cDM suggest combined contributions of β-cell loss, glucagon deficiency, and maldigestion (20). Additionally, shifts in bile acids and SCFA pools emphasize the contribution of gut–liver–pancreas crosstalk to glucose dysregulation (21). Current hypotheses propose a bidirectional axis in which microbial metabolites, particularly SCFA, influence pancreatic exocrine and endocrine function, while pancreatic secretions reciprocally shape the composition and activity of the gut microbiota. Pancreatic injury is associated with characteristic microbial shifts—including enrichment of *Escherichia*—barrier dysfunction and bacterial translocation, with the severity of these alterations correlating with both EPI and systemic complications (1, 22). However, it remains unclear whether gut microbial signatures predominantly reflect exocrine pancreatic dysfunction or the metabolic consequences of islet cell loss in T3cDM. Distinguishing microbiome changes driven by exocrine dysfunction versus broader metabolic disturbances can guide supportive strategies: if dysbiosis is chiefly EPI-related, microbiota-directed measures (dietary modulation, pre-/probiotics) may be considered as adjuncts to optimized PERT and nutritional care in the future.

Accordingly, we characterized microbiome and metabolome alterations in established T3cDM, comparing T3cDM with T1DM longitudinally and using in silico and machine learning approaches to predict microbial metabolic exchanges and link them to clinical outcomes. We found marked *Escherichia–Shigella* enrichment in T3cDM that tracked with pancreatic functional loss and EPI rather than T1DM-related metabolic changes, alongside distinct microbial lipid and carbohydrate production profiles, supporting a model in which the gut microbiome acts as a dynamic metabolite pool that senses and modulates pancreatic dysfunction.

## 2 Methods

### 2.1 Study population

We enrolled 48 participants: 21 with T1DM, 17with T3cDM, and 10 healthy controls with a follow up. Patients were recruited in 2018 at the Department of Internal Medicine I, Gastroenterology, Hepatology, Endocrinology and Metabolism, Medical University of Innsbruck. All T3cDM participants with evidence of EPI—based on symptoms or low fecal elastase—received PERT.

Definitions of T3cDM and T1DM followed previously published consensus criteria (23, 24). Healthy controls were selected based on the absence of comorbidities and no regular medication use. The T1DM and T3cDM group were 100% insulin dependent. The mean interval between baseline clinical and laboratory assessment and the second follow-up was 2411.1 (SD ± 323.8) days, and stool samples were obtained a mean of 571.2 (SD ± 339.0) days after baseline. Sex distribution did not differ significantly between groups. T1DM patients were significantly younger than both T3cDM and healthy participants, whereas smoking and daily alcohol intake > 20 g were more common in T3cDM. Within the T3cDM group, 13.0% (n = 3) had undergone total pancreatectomy, 39.1% (n = 9) partial pancreatectomy (pancreatic head or distal resection), and 47.8% (n = 5) had chronic pancreatitis.

### 2.2 Machine learning modelling

We trained LASSO (glmnet, mixture=1) logistic regression models using the tidymodels framework in R (v4.3.0) on genus-level microbial profiles derived from the nf-core/ampliseq pipeline (25, 26). Taxa were aggregated at the genus level, filtered (mean relative abundance >3%, prevalence >5%), and normalized. Samples were annotated into three groups (n=21 T1DM, n=17 T3cDM, n=10 healthy) based on metadata. Separate binary classification models were trained for each comparison, with cross-validation applied. For each comparison, data were split into training (70%) and independent test (30%) sets using stratified sampling to preserve class proportions. Model performance was evaluated by accuracy, AUC (area under the curve), and ROC (receiving operating curve) curves, while feature importance was inferred from non-zero model coefficients.

### 2.3 Microbial community modelling

Community metabolic modelling (CMM) was employed to integrate genome-scale metabolic models of individual microbial species, enabling the simulation and prediction of interspecies metabolic interactions, nutrient exchanges, and overall community function within a shared metabolic environment. CMM was performed with MICOM v0.25.1 (27). This analysis included 48 samples from patients stratified as: 21 with T1DM, 17 with T3cDM, and 10 healthy controls. Taxonomic profiles from nf-core/ampliseq outputs were collapsed to the genus level, filtering for relative abundance ≥2.5%. Community models were constructed with AGORA v1.03 genus-level metabolic models (28). Regarding the screening criteria for the AGORA model library taxa were included if their relative abundance was ≥0.0001 and a corresponding genus-level AGORA model was available. Simulations were performed using a predefined gut “Western diet” medium (western_diet_gut.qza, https://doi.org/10.5281/zenodo.3755182) selected to reflect the dietary background of the cohort. The maximum uptake flux was constrained to the value specified in the medium file (mmol·gDW^-1^·h^-1^) and oxygen availability restricted (EX_o2_m = 0.001 mmol·gDW^-1^·h^-1^). Parsimonious flux balance analysis (strategy = “pFBA”) was implemented in MICOM using the cooperative tradeoff algorithm (tradeoff = 0.3) and the GLPK solver. Only optimal solutions were retained; fluxes <1×10^-6^ were set to zero and normalized to 1 g dry weight biomass, enabling comparison across taxa and samples. Sample specific growth rates and exchange fluxes were computed. Group differences (H, T1DM, T3cDM) were assessed using pairwise t-tests with Benjamini–Hochberg FDR correction, and effect sizes were estimated using rank-biserial correlation. Log_10_-transformed community abundances were modeled using linear regression with disease group (T3cDM vs T1DM) as the main predictor and sex as a covariate. Regression coefficients, t-statistics, p-values, and FDR-adjusted p-values were computed, and metabolites were ranked by absolute t-value to illustrate the magnitude and direction of group differences. To explore clustering of metabolic variation by taxon or disease, t-distributed stochastic neighbor embedding (t-SNE) was applied to reduced flux profiles.

### 2.4 Ethical considerations

All research was conducted in accordance with both the Declarations of Helsinki and Istanbul. The institutional ethics commission (Ethics Commission of the Medical University of Innsbruck) approved the study protocol AN 1100/2025 and 1156/2018, written consent was given by all subjects.

### 2.5 Data availability

Microbiome sequencing data and statistical analysis results are available in Zenodo (https://doi.org/10.5281/zenodo.16794434). All scripts used for data analysis, statistics, and visualizations are provided through the GitHub repository (https://github.com/icbi-lab/diabetes_microbe).

More detailed information for material and methods is available in the supplementary data.

## 3 Results

### 3.1 Reduced diversity and distinct community structures in T3cDM compared with T1DM and healthy controls

Clinical characteristics of the cohorts are provided in **Supplementary Tables 1–3**.

**Figure 1:**
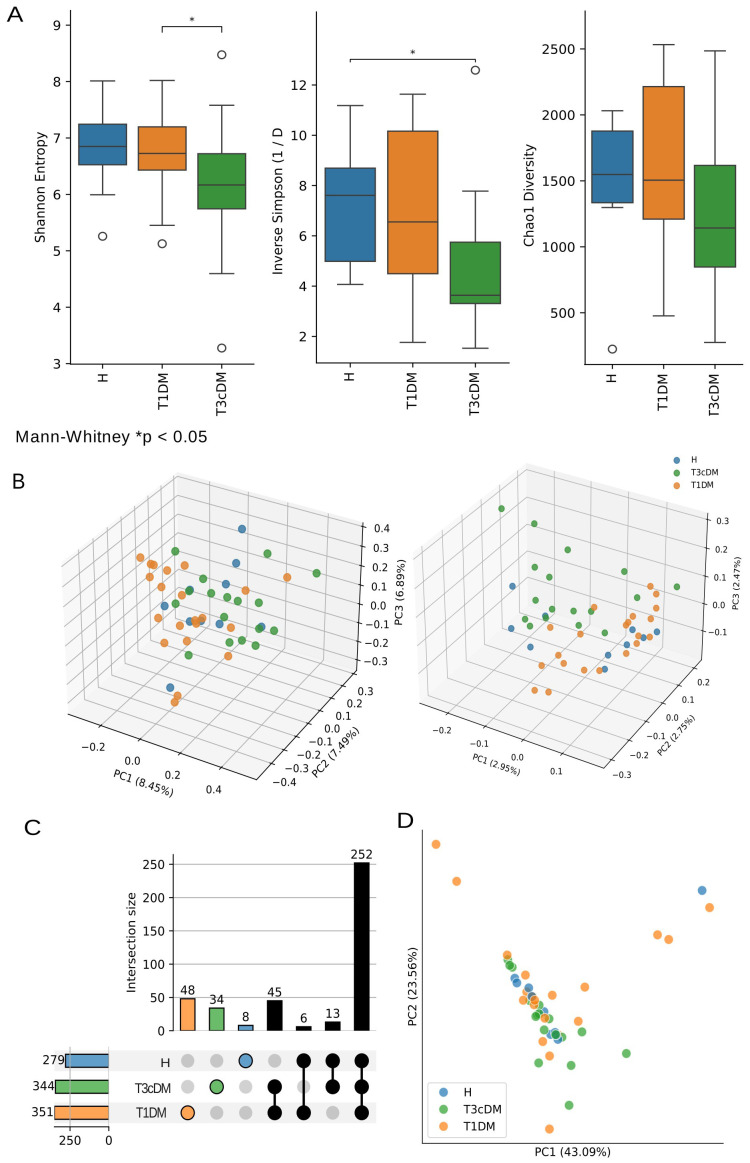
Bacterial taxa diversity and family overlap. **(A)** Community α-diversity analysis described by Shannon, Simpson and Chao1 index measurements, statistical annotation indicates p.adj < 0.05 after two-sided Mann Whitney test and FDR correction. **(B)** Community β-diversity analysis described by Bray-Curtis and Jaccard distances. All analyses are separated by condition: Healthy (H), T1DM (Type 1 Diabetes Mellitus), T3cDM (Type 3C Diabetes Mellitus). **(C)** UpSetPlot visualizing the set overlap for all the recorded families. **(D)** Principal component analysis (PCA) for all the samples where the two principal components account for most of the variance. Mann–Whitney test statistical annotation*p.val < 0.05.

Microbial diversity was significantly reduced in T3cDM compared with both T1DM and healthy controls, as shown across multiple α-diversity indices. The Simpson diversity index differed between healthy controls and T3cDM (p.adj < 0.05), and Shannon entropy differed between T1DM and T3cDM (p.adj < 0.05) (**Figure 1A**).

β-diversity analyses supported these observations. Bray–Curtis dissimilarity revealed separation between T1DM and T3cDM, with healthy controls clustering in between (**Figure 1B.1**). Jaccard-based PCoA provided clearer separation of T1DM and T3cDM, while T1DM partially overlapped with healthy controls (**Figure 1B.2**).

In total, 132 microbial families were detected, with 86 shared across all groups (**Figure 1C**). Twenty families were unique to T1DM and five to T3cDM. Principal component analysis showed greater dispersion in T1DM and T3cDM compared with controls, with pronounced heterogeneity in T1DM (**Figure 1D**).

### 3.2 Disease-specific shifts at the family level

**Figure 2:**
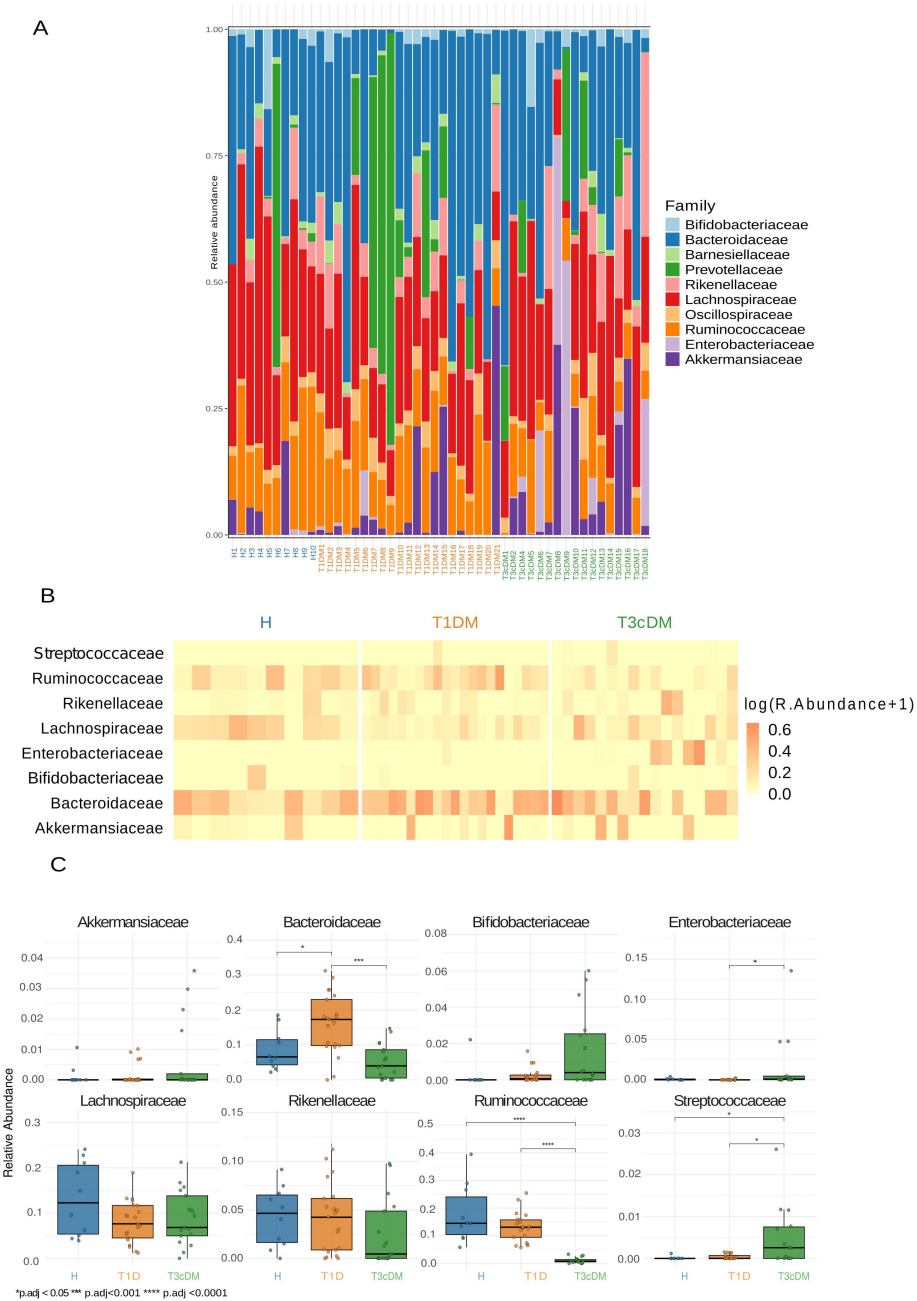
Family-level bacterial composition. **(A)** The stacked bar plot depicts the relative abundance for the 10 most abundant families in all samples. **(B)** Heatmap showing the log(relative abundance +1) for the core microbiome, defined as the bacterial families present in more than 50% of the samples. **(C)** Boxplots for the log(relative abundance +1) of the core microbiome families together with pairwise comparison across the disease groups using Mann–Whitney test statistical annotation. *p.adj < 0.05, *** p.adj < 0.001, **** p.adj<0.0001.

The ten most abundant families in T3cDM included Bifidobacteriaceae, Bacteroidaceae, Barnesiellaceae, Prevotellaceae, Rikenellaceae, Lachnospiraceae, Oscillospiraceae, Ruminococcaceae, Enterobacteriaceae, and Akkermansiaceae (**Figure 2A**), consistent with taxonomy assignments shown in the Sankey diagram (**Supplementary Figure 1**).

Core microbiome analysis identified eight families consistently shared across groups (**Figure 2B**). Pairwise comparisons of these core families revealed disease-specific differences (**Figure 2C**). Enterobacteriaceae and Streptococcaceae were significantly enriched in T3cDM compared with both T1DM and healthy controls. Bacteroidaceae were enriched in T1DM compared with the other groups, whereas Ruminococcaceae were enriched in healthy controls.

### 3.3 Logistic regression and LEfSe identify microbial signatures of T3cDM

**Figure 3:**
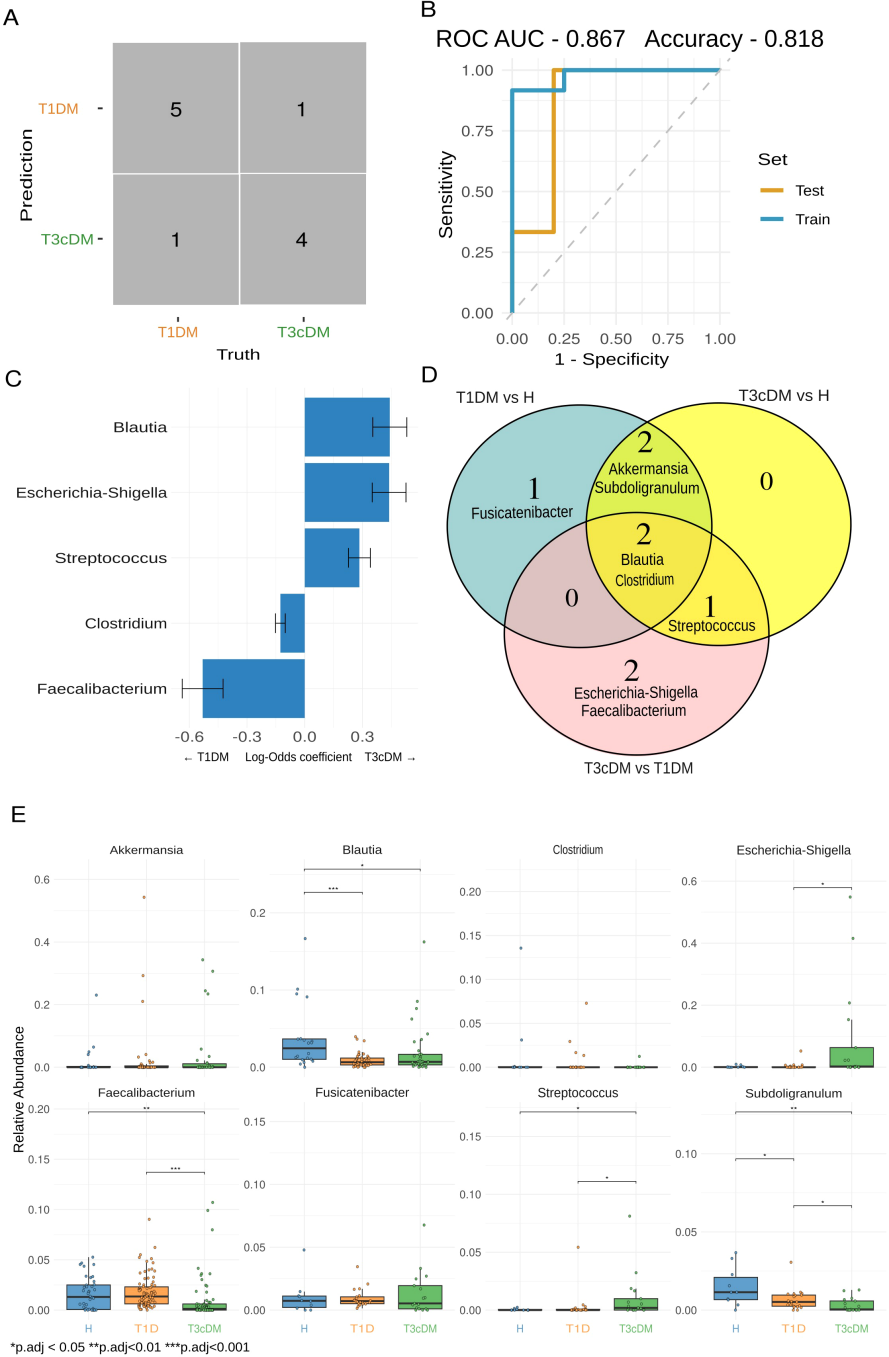
Microbial signatures identified by logistic regression. **(A)** Confusion matrix evaluating the performance of the microbial logistic regression model for the classification between T1DM and T3cDM on the test set. **(B)** Receiver operating characteristic (ROC) curve showing the performance of the classification model. Visualizes the relationship between the true positive rate (sensitivity) and the false positive rate (1 - specificity). The model achieved an area under the ROC curve (AUC) of 0.867 and an accuracy of 0.818 on the test set. **(C)** Bar plot showing the top five genera contributing to the classification model based on their log-odds coefficient. Positive coefficients indicate genera associated with increased odds of classification into the T3cDM, while negative coefficients suggest association with T1DM. **(D)** Venn diagram showing the overlap of the top five genera from pairwise classification models comparing T1DM vs. T3cDM, T1DM vs. H, and T3cDM vs. H. **(E)** Boxplots plots showing relative abundance of microbial genera identified as top contributors in the logistic regression model from all the pairwise classification models. Mann–Whitney test statistical annotation *p.adj < 0.05, ** p.adj < 0.01, ***p.adj < 0.001.

To identify discriminatory taxa, logistic regression was applied at the genus level. For T1DM vs. T3cDM (primary comparison), the confusion matrix showed robust classification with a ROC AUC of 0.867 and accuracy of 0.818 in the test set (**Figures 3A, B**). The most influential genera included *Blautia, Escherichia–Shigella, Streptococcus, Clostridium*, and *Faecalibacterium* (**Figure 3C**). Overlap across models was visualized by Venn diagram (**Figure 3D**), and relative abundances confirmed that *Blautia, Faecalibacterium*, and *Subdoligranulum* were depleted in both diabetes groups, whereas *Streptococcus* and *Escherichia–Shigella* were enriched in T3cDM (**Figure 3E**).

LEfSe analysis confirmed discriminatory genera across pairwise comparisons (**Supplementary Figure 2**), overlapping with taxa identified by regression models. *Faecalibacterium prausnitzii* and *Subdoligranulum* were more abundant in healthy controls than in T1DM, whereas *Streptococcus* and *Escherichia–Shigella* were enriched in T3cDM.

Sex-specific analyses further revealed distinct microbial signatures (**Supplementary Figure 3**): *Turicibacter* was enriched in T1DM males, *Blautia* in T1DM females. In T3cDM, *Ruminococcus* and *Subdoligranulum* were enriched in males while *Eggerthella*, *Eubacterium*, and *Negativibacillus* were enriched in females.

Further analyses included model validation across all pairwise comparisons (**Supplementary Material**). Confusion matrices demonstrated good classification performance for T1DM vs. controls and T3cDM vs. controls (**Supplementary Figure 4**). ROC curves yielded AUCs of 0.867 (accuracy 0.75) for T1DM vs. controls, and 0.833 (accuracy 0.70) for T3cDM vs. controls (**Supplementary Figure 5**). The top five genera contributing to classification were visualized for both comparisons (**Supplementary Figure 6**).

Correlation analyses highlighted strong clinical associations. *Escherichia–Shigella* abundance correlated closely with pancreatectomy (**Supplementary Figure 7**). Its abundance was significantly higher in individuals with pancreatectomy, but did not differ between left, right, or total resections (**Supplementary Figure 8**). Furthermore, *Escherichia–Shigella* abundance was significantly correlated with stool elastase, the most reliable marker of EPI (**Supplementary Figure 9**).

Longitudinal clinical changes were evaluated by generalized linear mixed-effects models (**Supplementary Figure 10**). In T1DM, NT-proBNP, alkaline phosphatase, high-sensitivity troponin T, and body-mass index (BMI) increased over a 5-year period, whereas in T3cDM, LDL cholesterol decreased modestly. Furthermore, BMI did not differ significantly between groups (p = 0.896), but metabolic dysfunction-associated steatotic liver disease (MASLD), coronary artery disease (CAD), and arterial hypertension were significantly more prevalent in T3cDM (p = 0.015, p = 0.014, and p < 0.001, respectively). Use of antihypertensive agents and diuretics was also more frequent in T3cDM (p < 0.001 and p = 0.019). Detailed characteristics are provided in **Supplementary Tables 1–3**.

### 3.4 Functional modeling reveals altered growth dynamics and metabolite flux in T3cDM

**Figure 4:**
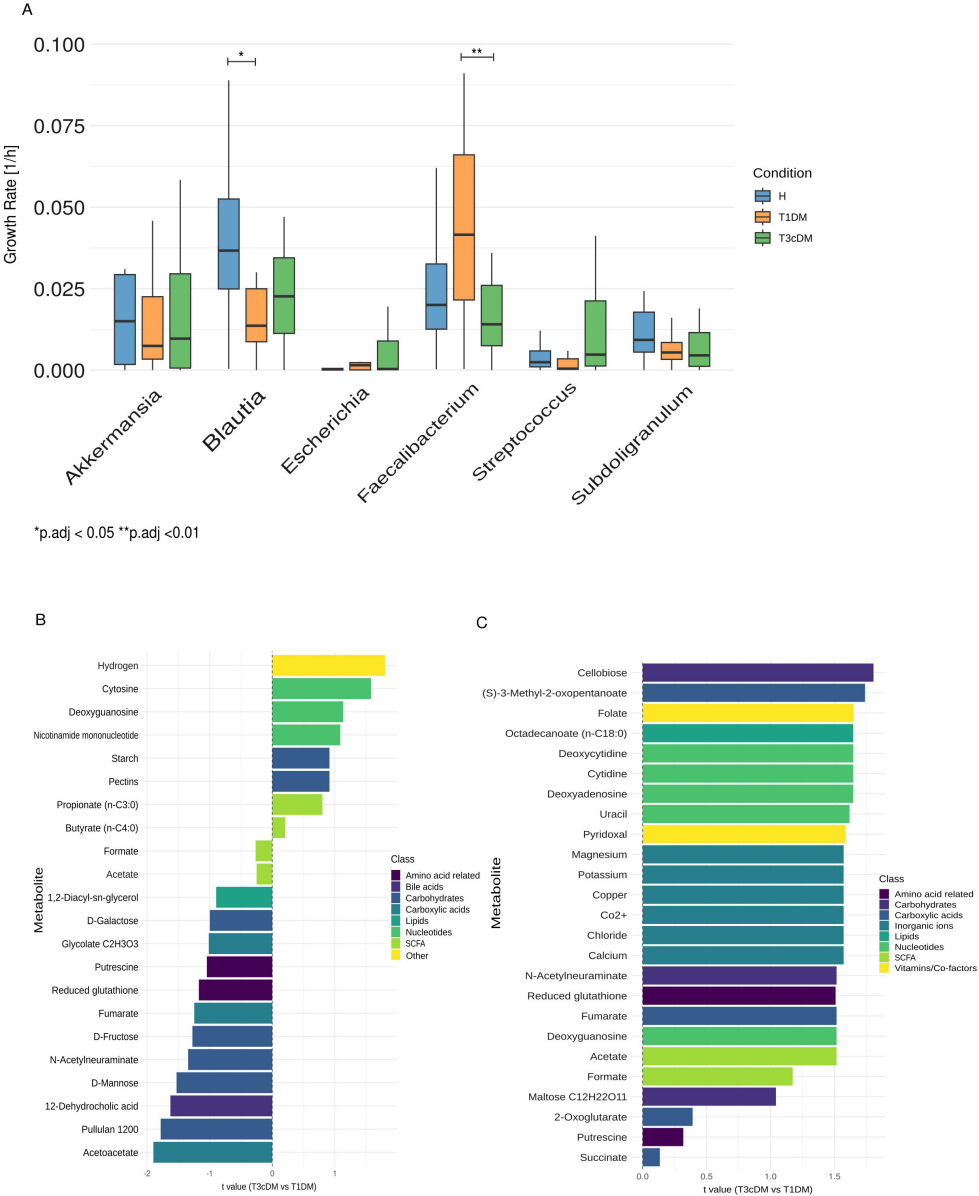
Microbial community model metabolic in-silico prediction. **(A)** Boxplots showing the predicted growth rates of bacterial genera previously identified by logistic regression. **(B)** Barplots showing linear regressing analysis of metabolites abundance for all identified genera and exclusively for Escherichia-Shigella **(C)** between T3cDM vs T1DM, adjusted for sex as confounder. Positive t value indicated higher concentrations in T3cDM and color code indicates metabolite class. Significance was defined as *p*.adj < 0.05 (Wilcoxon test).

Community metabolic modelling revealed functional perturbations underlying compositional shifts. Flux-based clustering further resolved microbial functional differences (**Supplementary Figure 11**). *Akkermansia* and *Blautia* were predominantly associated with T1DM, *Escherichia–Shigella* with T3cDM, whereas *Faecalibacterium*, *Streptococcus*, and *Subdoligranulum* showed more variable distributions. Predicted genus-specific growth rates from community metabolic simulations are shown for the 20 most abundant genera (**Supplementary Figure 12**) and indicated clear genus-level differences (**Figure 4A**; **Supplementary Figure 12**). *Escherichia–Shigella* had the highest growth rate in T3cDM and the lowest in healthy controls. *Blautia* growth was reduced in T1DM, and *Faecalibacterium prausnitzii* growth was lowest in T3cDM.

Predicted metabolite abundances revealed enrichment of nucleotides (cytosine, deoxyguanosine, nicotinamide mononucleotide), carbohydrates (starch, pectins), hydrogen, and SCFA (propionate, butyrate) in T3cDM (**Figure 4B**). In contrast, several carbohydrates (D-fructose, D-mannose, N-acetylneuraminate), amino acid–related metabolites (putrescine, glutathione), lipids, carboxylic acids, bile acids, and SCFA (acetate, formate) were depleted. In more detail, we can see connected genera of interest to metabolite predicted abundance (**Supplementary Figure 13**). *Akkermansia*, *Blautia*, *Faecalibacterium*, and *Subdoligranulum* are connected to 2-oxobutanoate, D-galactose, N-acetylneuraminate, sodium, cellobiose, D-fructose, N-acetyl-D-glucosamine, D-glucuronate, glycerol, acetaldehyde, butyrate, acetate, ammonium, and calcium. *Escherichia–Shigella* and *Streptococcus* are linked to formate, 2-oxoglutarate, choline, trehalose, adenosine, chloride, copper, and CO_2_. Linear regression further identified *Escherichia–Shigella* as uniquely associated with a broad metabolite spectrum including inorganic ions, nucleotides, amino acid–related metabolites, carbohydrates, carboxylic acids, and vitamins (**Figure 4C**).

Together, these findings demonstrate that functional modeling resolves disease-specific metabolic alterations not evident from taxonomic profiles alone.

## 4 Discussion

There is broad consensus that both endocrine and exocrine pancreatic insufficiency are linked to intestinal dysbiosis, manifesting as altered microbial composition and reduced α-diversity. However, causal relationships in humans remain elusive, and the downstream effects of dysbiosis on host immunity are only partially understood.

Our findings extend prior observations of microbiome alterations in chronic pancreatitis, post-pancreatectomy states, and T1DM by demonstrating that T3cDM—in which endocrine failure follows exocrine loss—exhibits a particularly pronounced dysbiosis marked by reduced diversity and selective enrichment of facultative pathobionts such as Enterobacteriaceae and Streptococcaceae (29–33). The resemblance of these shifts to those seen in chronic pancreatitis, pancreatic cancer, and EPI—settings characterized by impaired digestion, malabsorption, and a propensity for SIBO—supports a central role for exocrine dysfunction in restructuring gut ecology (29, 32, 34). In contrast, T1DM shows preserved α-diversity and enrichment of immune-associated taxa, underscoring the distinctiveness of the T3cDM microbial signature (34). The low diversity and loss of SCFA-producing genera (*Blautia, Faecalibacterium, Subdoligranulum*), alongside enrichment of *Streptococcus* and *Escherichia–Shigella*, suggest erosion of protective functions and expansion of opportunists (32, 35, 36); notably, *Enterobacteriaceae* growth coincided with reduced predicted growth of *Blautia* and *Faecalibacterium*, and *Escherichia–Shigella* was highest in patients with low stool elastase, implicating EPI as a key ecological driver. Importantly, EPI cannot—and should not—be separated from metabolic disturbances: exocrine failure alters nutrient sensing, bile acid signaling, and microbial SCFA profiles, thereby modulating incretin release and systemic metabolism; conversely, PERT interfaces with both digestion and metabolic regulation, offering a plausible mechanism for the observed microbial shifts (34, 37). Experimental data reinforce this interpretation: enzyme deficiency promotes *Enterobacteriaceae* expansion and is mitigated by pancreatic enzyme replacement therapy (PERT) in humans (37). In parallel, controlled animal models demonstrate that exocrine pancreatic insufficiency reduces microbial α-diversity and reshapes community composition, and that enzyme supplementation (PERT or lipase) partially restores a healthier microbial configuration; these microbiota alterations rapidly reappear when enzymes are withdrawn and reverse again upon reintroduction (37–39).

Our data indicate that exocrine dysfunction in T3cDM is a dominant ecological driver of community collapse and pathobiont enrichment within an inseparable exocrine–metabolic axis, underscoring the need for trials that integrate microbiome-informed phenotyping with optimized PERT and metabolic control and evaluate adjunct microbiome-directed therapies.

Metabolomic profiling, although the sample size was a limiting factor, revealed a distinct metabolic signature in T3cDM, characterized by reduced metabolite diversity, a dominance of hydrogen and nucleotide pathways, and limited SCFA and carbohydrate contributions. By contrast, T1DM samples displayed higher representation of carbohydrate, amino acid, and carboxylic acid pathways but similarly showed diminished SCFA levels, consistent with altered fermentation in autoimmune diabetes (35). Nucleotide enrichment in T3cDM suggests accelerated microbial turnover, while elevated polysaccharide residues likely reflect impaired enzymatic hydrolysis in the context of EPI (32, 34). Hydrogen production, driven by fermentation of undigested carbohydrates, was a dominant feature, with *Escherichia–Shigella* contributing metabolites such as formate and acetaldehyde that can compromise epithelial barrier integrity (40). The loss of butyrate-producing taxa further deprives the host of key microbial metabolites essential for maintaining epithelial and immune homeostasis (37–41).

Moreover, enhanced microbial metabolism of choline and glycine betaine may exacerbate cardiometabolic risk through the generation of trimethylamine (TMA) and its hepatic oxidation product, trimethylamine-N-oxide (TMAO) (42–44). These microbial–host co-metabolites are well-established mediators of atherogenesis and metabolic dysfunction, and their enrichment in T3cDM supports a mechanistic link between exocrine loss, dysbiosis, and systemic metabolic derangements.

Clinically, this is relevant because a considerable symptom burden may persist despite standard-of-care therapy, motivating evaluation of adjunct, symptom-oriented strategies that complement optimized PERT.

Although our cohort size was limited, we implemented supervised machine learning models to explore genus-level and metabolic features distinguishing T3cDM, T1DM, and healthy individuals. Recent studies have applied similar approaches to identify microbiome-derived signatures across pancreatic and metabolic diseases. Tan et al. and Yuan et al. integrated gut microbiota, serum metabolites, and lipidomic data in T1DM, revealing multi-omic biomarkers predictive of disease progression (35). Lee et al. and other groups have used machine learning to define microbiome-based diagnostic classifiers in pancreatic cancer and chronic pancreatitis, underscoring the diagnostic potential of AI-driven models (45–47). While these tools hold significant promise, it is essential to apply rigorous cross-validation, avoid data leakage, and interpret model features biologically rather than solely statistically (46). Our results, in line with these studies, highlight the emerging role of artificial intelligence in delineating microbiome–host interactions and identifying mechanistic pathways in metabolic and pancreatic diseases. Here, we used supervised models in an exploratory, hypothesis-generating mode to surface discriminative features, laying groundwork for future clinically oriented tools, and we envision biomarker-focused studies with external validation and pancreatic disease control cohorts without dysglycemia to robustly disentangle effects of pancreatic pathology from those of glucose metabolism.

In T3cDM, depletion of SCFA producers with enrichment of facultative pathobionts likely compromises epithelial barrier function and amplifies systemic inflammation: SCFAs such as butyrate and propionate support epithelial integrity and Treg homeostasis and inhibit HDAC/NF-κB signaling (36, 41), so their loss may facilitate translocation of microbial products and TLR/NLRP3-mediated immune activation, while Escherichia–Shigella metabolites (e.g., acetaldehyde, formate) further disrupt tight junctions and induce oxidative stress (40). Our integrative microbiome–metabolome analysis reveals a distinct dysbiotic and metabolic signature most consistent with exocrine insufficiency and maldigestion as dominant ecological drivers, yet inseparable from systemic metabolic disturbances within a shared exocrine–metabolic continuum.

The enrichment of facultative pathobionts, particularly *Escherichia–Shigella* and *Streptococcaceae*, together with the depletion of SCFA-producing genera such as *Blautia* and *Faecalibacterium*, defines a microbial ecosystem conducive to metabolic dysregulation, epithelial barrier disruption, and systemic inflammation. However, the cross-sectional nature of the analysis precludes causal inference. Optimized EPI management—including PERT—remains the primary therapy for the exocrine component of T3cDM. Microbiota-directed strategies should be explored only as adjuncts, ideally in trials that first standardize PERT optimization and then test whether dietary modulation or targeted microbiome interventions confer additional benefits in symptoms, nutritional status, and metabolic outcomes.

Longitudinal and interventional studies—especially those incorporating pancreatic enzyme replacement therapy, dietary modulation, or targeted microbiome interventions—are needed to clarify causal relationships between dysbiosis, exocrine dysfunction, and metabolic outcomes. Future integrative approaches combining metagenomic, transcriptomic, and immune-phenotyping data will be essential to identify strain-level microbial effectors driving host–microbe interactions. As a future outlook, mechanistic experiments would further complement the present study, particularly controlled *in vitro* epithelial co-culture systems designed to evaluate the impact of Escherichia–Shigella–derived metabolites on intestinal barrier function, including effects on tight junction protein expression (e.g., ZO-1, occludin) and epithelial permeability. In parallel, robust artificial intelligence frameworks with external validation should be employed to enhance biomarker discovery, refine predictive modeling, and advance microbiome- and metabolome-informed diagnostics and therapeutics in T3cDM and related disorders (45–47).

## Data Availability

The datasets presented in this study can be found in online repositories. The names of the repository/repositories and accession number(s) can be found in the article/**Supplementary Material**.

## Appendix

### Evidence before this study

We searched PubMed up to September 2025 for studies examining the gut microbiome in pancreatogenic diabetes (T3cDM). While numerous studies have reported microbial dysbiosis in chronic pancreatitis, pancreatic cancer, post-pancreatectomy patients, and type 1 diabetes (T1DM), data in T3cDM are limited. Most prior work focused on cross-sectional associations between microbial composition and metabolic parameters, with few studies integrating functional metabolomics or mechanistic modelling.

### Added value of this study

Our study provides a comprehensive, longitudinal comparison of gut microbiota and microbial metabolic function in T3cDM, T1DM, and healthy controls. Using community metabolic modelling, we identify a distinct microbial and metabolomic signature in T3cDM, characterized by enrichment of *Escherichia–Shigella* and Streptococcaceae, depletion of short-chain fatty acid (SCFA)-producing genera, and metabolic shifts such as increased hydrogen and nucleotide production. These alterations correlate with exocrine pancreatic insufficiency rather than endocrine dysfunction, highlighting EPI as a key driver of gut microbial dysbiosis and metabolite perturbation.

Our findings support a mechanistic gut–pancreas axis in T3cDM, linking EPI to microbial and metabolic changes that may contribute to chronic inflammation and cardiometabolic risk. Integration of microbial and metabolomic data offers potential for novel diagnostic biomarkers and therapeutic strategies targeting the microbiome to mitigate complications in T3cDM.

## Glossary

ASVs: Amplicon sequence variants
ANOVA: Analysis of variance
BMI: Body mass index
CAD: Coronary artery disease
EPI: Exocrine pancreatic insufficiency
Fe^3+^: Iron
GLMM: Generalized linear mixed-effects models
GM: Gut microbiome
H: Healthy control
IBD: Inflammatory bowel diseases
IQR: Inter-quartile range
LEfSe: Linear discriminant analysis Effect Size
MASLD: Metabolic dysfunction-associated steatotic liver disease
NODAP: New-onset diabetes after pancreatitis
PCR: Polymerase chain reaction
T3cDM: Pancreoprivic diabetes
ROC: Receiver operating characteristic
SCFA: Short-chain fatty acids
SD: Standard deviation
TMA: Trimethylamine
TMAO: Trimethylamine N-oxide
T1DM: Type 1 Diabetes
