# Supplementary material for: Escherichia-Shigella expansion and metabolite dysregulation in type 3c diabetes: linking microbiome alterations to exocrine pancreatic insufficiency
Source: Front Endocrinol (Lausanne). 2026 Mar 27;17:1786756. doi: 10.3389/fendo.2026.1786756 (PMC13065728; doi:10.3389/fendo.2026.1786756)
Supplement: Supplementary file 1 [file DataSheet1.pdf]

Escherichia-Shigella Expansion and metabolite dysregulation in type 3c diabetes: linking microbiome alterations to exocrine pancreatic insufficiency

Erika Kvalem Soto[a*]; Valeria Wagner[b*]; Julia Engl[b,c]; Michael Mederer[b]; Veronika Cibulkova[b]; Johanna Piater[b] , Benedikt Schäfer[b], Elena Dunzendorfer[b]; Silvio Waschina[d]; Susanne Kaser[b]; Konrad Aden[e,f], Zlatko Trajanoski[a]; Herbert Tilg[b]; Maria Effenberger[b]

[a] Biocenter, Institute of Bioinformatics, Medical University of Innsbruck, Innsbruck, Austria

[b] Department of Internal Medicine I, Gastroenterology, Hepatology, Endocrinology & Metabolism, Medical University of Innsbruck, 6020 Innsbruck, Austria

[c] Department of Medicine, Hospital of Brixen (SABES-ASDAA), Bessanone-Brixen, Italy; Teaching Hospital of Paracelsus Medical University

[d] Institute for Human Nutrition and Food Science, Division Nutriinformatics, Christian-Albrechts-University of Kiel, Kiel, Germany

[e] Institute of Clinical Molecular Biology, Christian-Albrechts-University and University Hospital Schleswig-Holstein, Campus Kiel, 24105 Kiel, Germany

[f] Department of Internal Medicine I., Christian-Albrechts-University and University Hospital Schleswig-Holstein, Campus Kiel, 24105 Kiel, Germany

Table of contents

- **Supplementary figure legend**
- **Supplementary figures**
- **Supplementary tables**
- **Additional methods**
- **References**

Supplementary figure legend

Figure S1: Sankey plot showing gut microbiome composition across taxonomic ranks from kingdom (left) to genus (right).

Figure S2: Barplot showing Linear discriminant analysis Effect Size (LEfSe) pairwise disease group comparisons. Bars depict LDA effect size for features identified by LEfSe (Kruskal–Wallis p.value <0.05, LDA ≥ 2)

Figure S3: Barplot showing Linear discriminant analysis Effect Size (LEfSe) analysis for the comparison female vs. male within each disease group (Kruskal–Wallis p.value <0.05, LDA ≥ 2). Distinct sex-related microbial patterns were detected in all groups, including healthy controls, suggesting that disease status modifies sex–microbiota interactions. In T1DM, *Turicibacter* was enriched in males, while *Blautia* was enriched in females. In T3cDM, males showed higher abundances of *Ruminococcus* and *Subdoligranulum*, whereas females exhibited enrichment of *Eggerthella*, *Eubacterium*, and *Negativibacillus*.

Figure S4: Confusion matrix of the performance of the microbial logistic regression model on the test set for (A) T1DM vs. healthy controls (B) T3cDM vs. healthy controls. The matrix describes from left to right true positives, false negatives, false positives, and true negatives.

Figure S5: Receiver-operating characteristic (ROC) curves for classifiers distinguishing (A) T1DM from healthy controls and (B) T3cDM from healthy controls, shown for training and test sets. Curves plot sensitivity versus 1–specificity; the diagonal indicates chance. Test-set performance: (A) AUC = 0.867, accuracy = 0.75; (B) AUC = 0.833, accuracy = 0.70.

Figure S6: Top five genera contributing to the classification model, ranked by log-odds ratios extracted from non-zero LASSO coefficients for the comparison (A) T1DM vs. healthy controls (B) T3cDM vs. healthy. Positive (right) coefficients mark genera linked to higher odds of the model's positive class; negative (left) coefficients indicate association with the model's negative class.

Figure S7: Heatmap of Pearson correlation coefficients between genus-level relative abundance and clinical variables at baseline (BS). Tile color encodes Pearson coefficient (r) where red means positive and purple mean negative. Statistical annotation: *p.adj < 0.05, ** p.adj < 0.01, *** p.adj < 0.001.

Figure S8: Bosplot of *Escherichia–Shigella* relative abundance (x) versus pancreatectomy status. Dots show pancreatectomy resection types (Partial resection left, Partial resection right, and Resection). Each dot represents one patient. Boxes denote median and IQR; whiskers are 1.5×IQR. Mann-Whitney-Test *p.value < 0.05 **p.value > 0.01.

Figure S9: Scatterplot depicting the relationship between *Escherichia–Shigella* relative abundance (x-axis) and stool elastase levels (y-axis). The green line denotes the linear regression fit with 95% confidence intervals. A positive regression slope ($\beta > 0$; $P = 0.038$) indicates that increased *Escherichia–Shigella* abundance is associated with reduced stool elastase concentrations, consistent with exocrine pancreatic insufficiency (EPI).

Figure S10: Forest plot of clinical and laboratory variables at baseline and follow-up, analyzed using generalized linear mixed-effects models (GLMM). In T1DM, NT-pro-BNP, alkaline phosphatase, high-sensitivity troponin T, and BMI significantly increased over time, whereas in T3cDM LDL cholesterol showed a modest but significant decrease. Points show effect estimates, odds ratios for binary outcomes. Horizontal bars indicate 95% CIs; the vertical dashed line at 0 marks no change. Statistical annotation: *p.adj < 0.05, ** p.adj < 0.01, *** p.adj < 0.001.

Figure S11: Two-dimensional t-SNE embedding of predicted community flux vectors, colored by (A) taxa (B) disease group (T1DM, T3DM, H). Distances are computed on predicted community flux vectors and proximity reflects similarity of predicted metabolic outputs. Selected genera are previously identified by logistic regression.

Figure S12: Dot plot of model-predicted genus-specific growth rate from community metabolic simulations for T1DM, T3cDM and H. The x-position indicates the predicted growth rate of the indicated genus. Selected genera are the top 20 genera from the community metabolic simulation ranked by growth rate.

Figure S13: Sankey diagram showing the connections between the bacteria genera of interest with their associated metabolites. Wider links indicate higher predicted metabolite abundance.

Supplementary figures

Figure S1

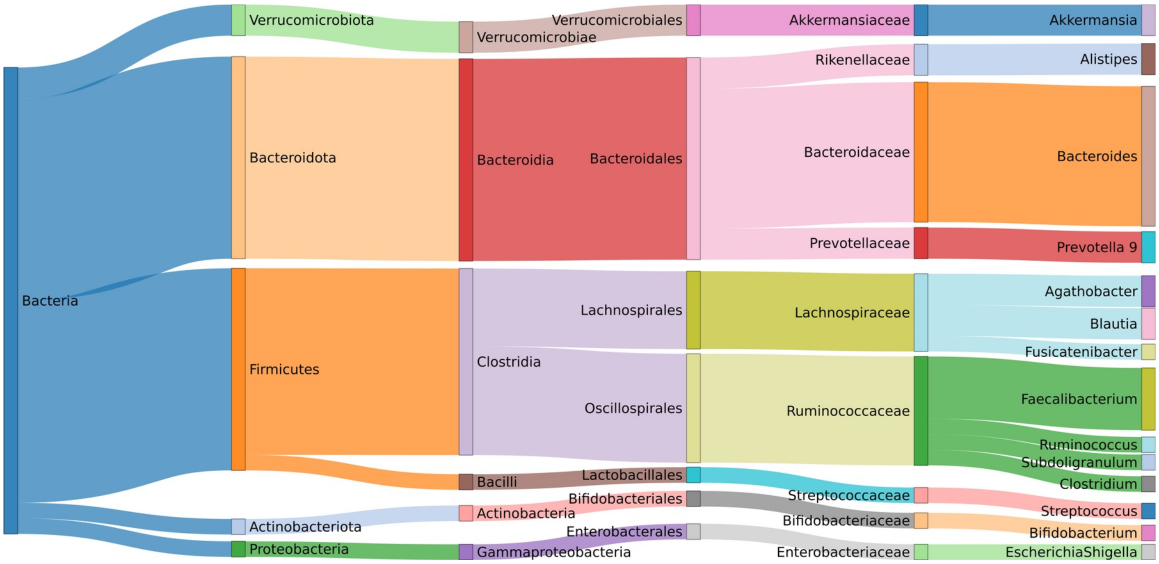

Figure S2

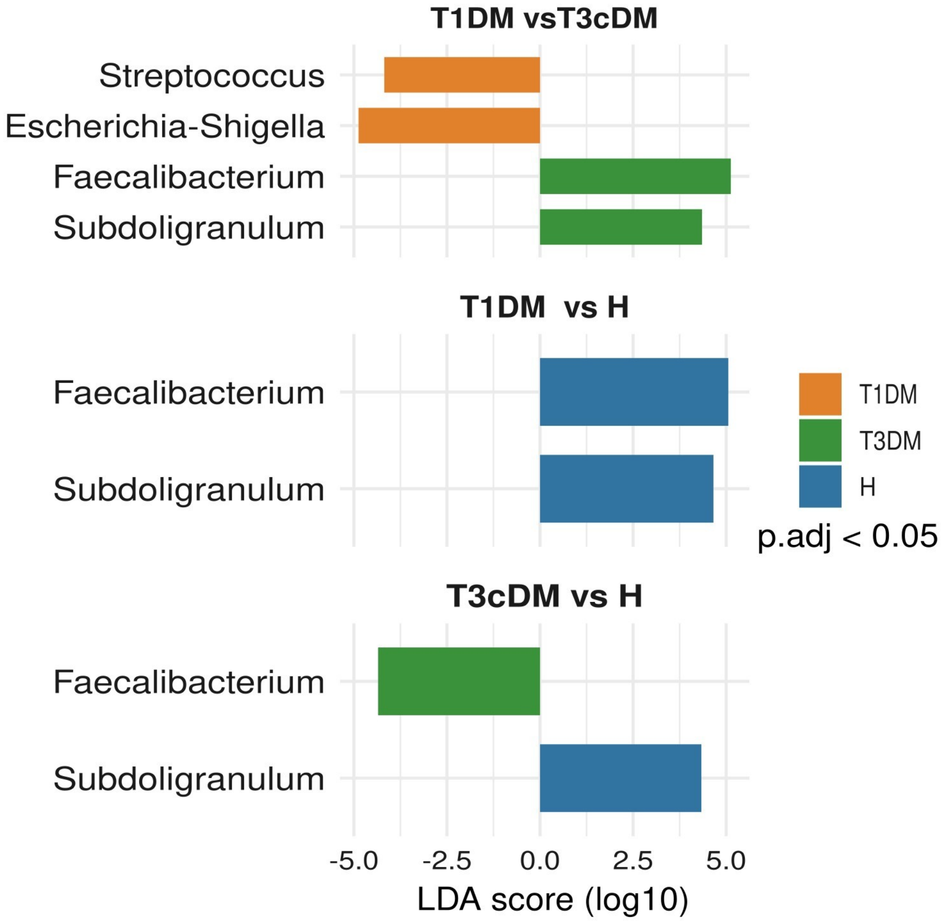
T1DM vsT3cDM
Streptococcus
Escherichia-Shigella
Faecalibacterium
Subdoligranulum
T1DM vs H
Faecalibacterium
Subdoligranulum
T1DM
T3DM
H
p.adj < 0.05
T3cDM vs H
Faecalibacterium
Subdoligranulum
-5.0
-2.5
0.0
2.5
5.0
LDA score (log10)

Figure S3

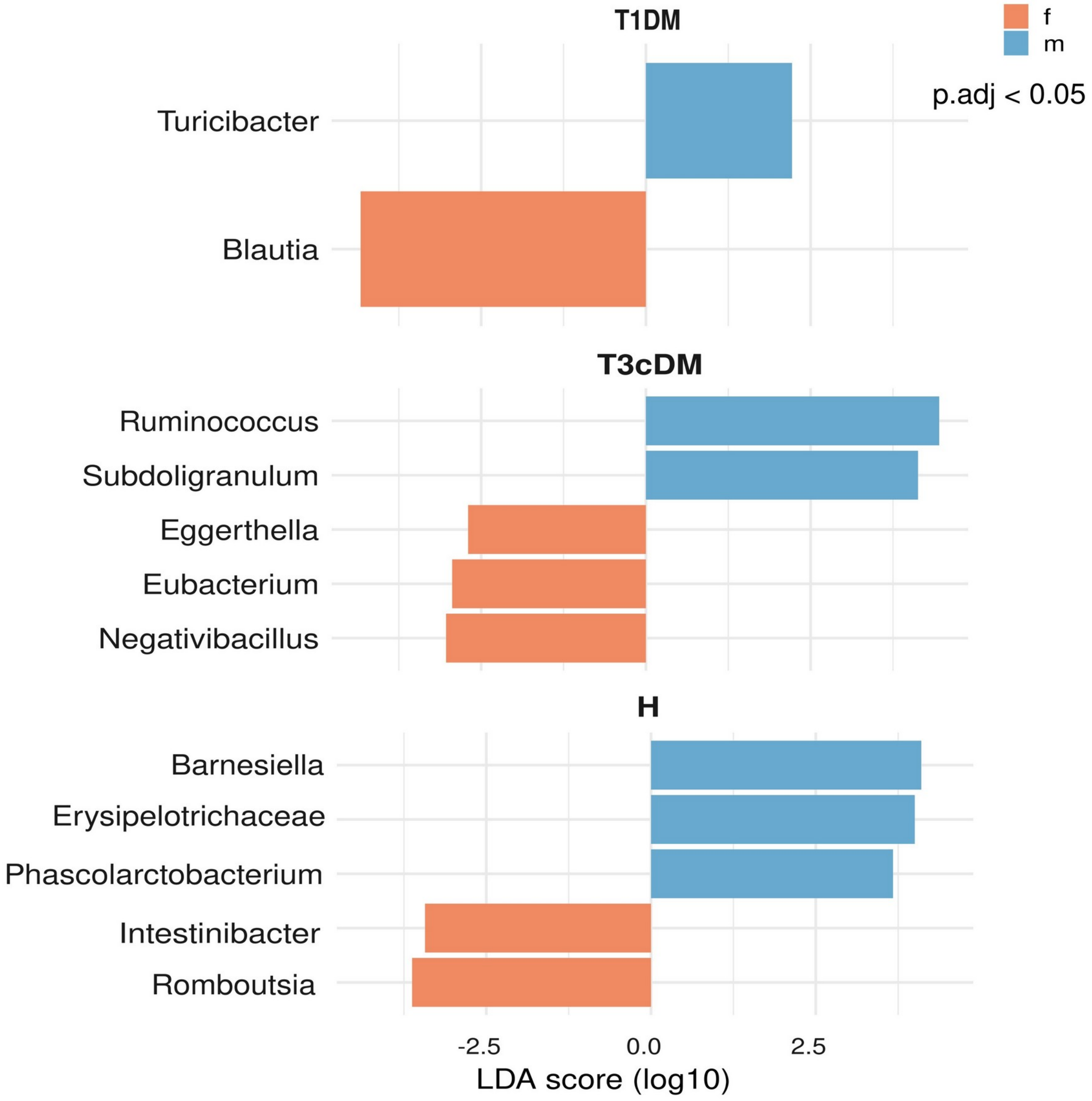
T1DM
f
m
p.adj < 0.05
Turicibacter
Blautia
T3cDM
Ruminococcus
Subdoligranulum
Eggerthella
Eubacterium
Negativibacillus
H
Barnesiella
Erysipelotrichaceae
Phascolarctobacterium
Intestinibacter
Romboutsia
-2.5
0.0
2.5
LDA score (log10)

Figure S4

A

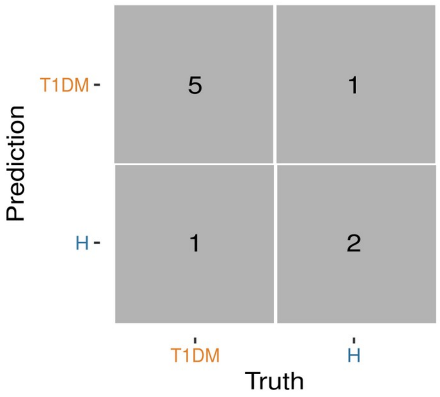
Prediction
T1DM
H
5
1
1
2
T1DM
H
Truth

B

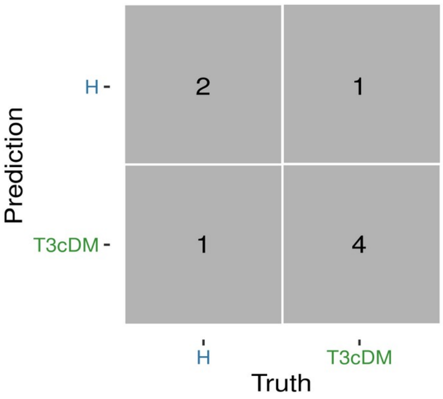
Prediction
H
T3cDM
2
1
1
4
H
T3cDM
Truth

Figure S5

A

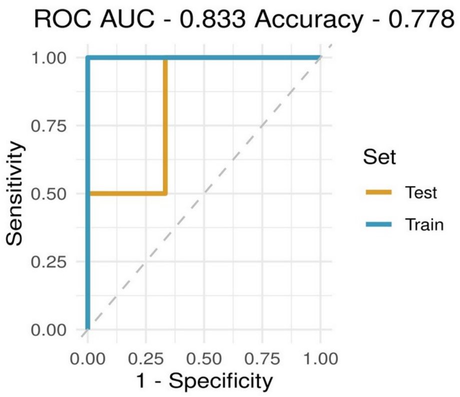
ROC AUC - 0.833 Accuracy - 0.778
1.00
0.75
0.50
0.25
0.00
Sensitivity
0.00 0.25 0.50 0.75 1.00
1 - Specificity
Set
Test
Train

B

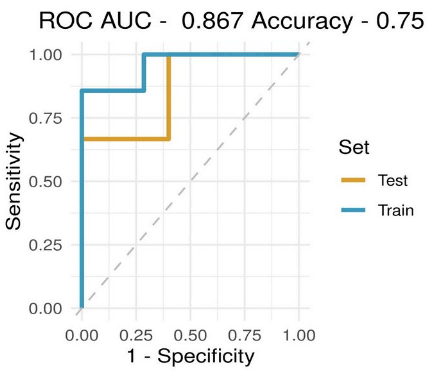
ROC AUC - 0.867 Accuracy - 0.75
1.00
0.75
0.50
0.25
0.00
Sensitivity
0.00 0.25 0.50 0.75 1.00
1 - Specificity
Set
Test
Train

Figure S6

A B

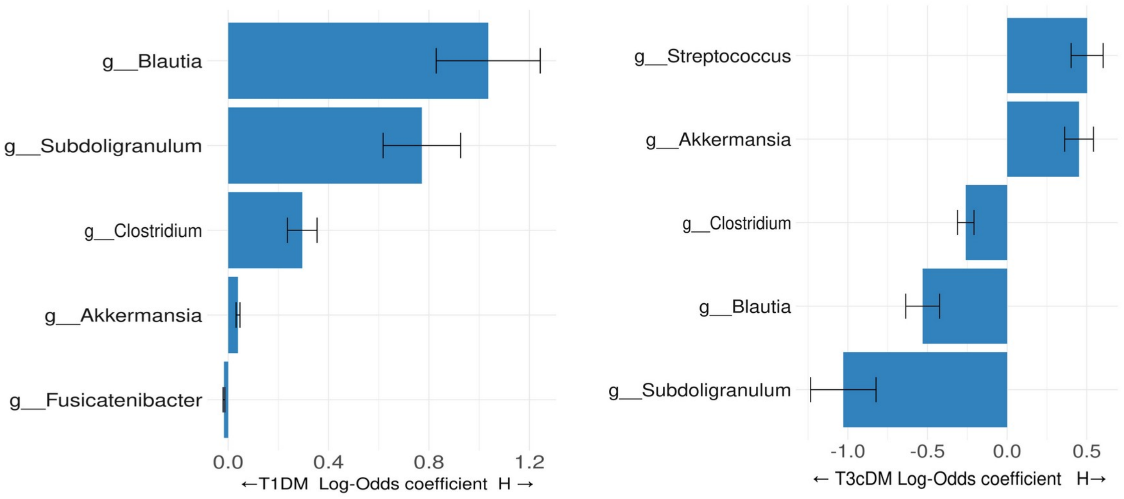
g__Blautia
g__Subdoligranulum
g__Clostridium
g__Akkermansia
g__Fusicatenibacter
0.0
0.4
0.8
1.2
←T1DM Log-Odds coefficient H →
g__Streptococcus
g__Akkermansia
g__Clostridium
g__Blautia
g__Subdoligranulum
-1.0
-0.5
0.0
0.5
← T3cDM Log-Odds coefficient H→

Figure S7

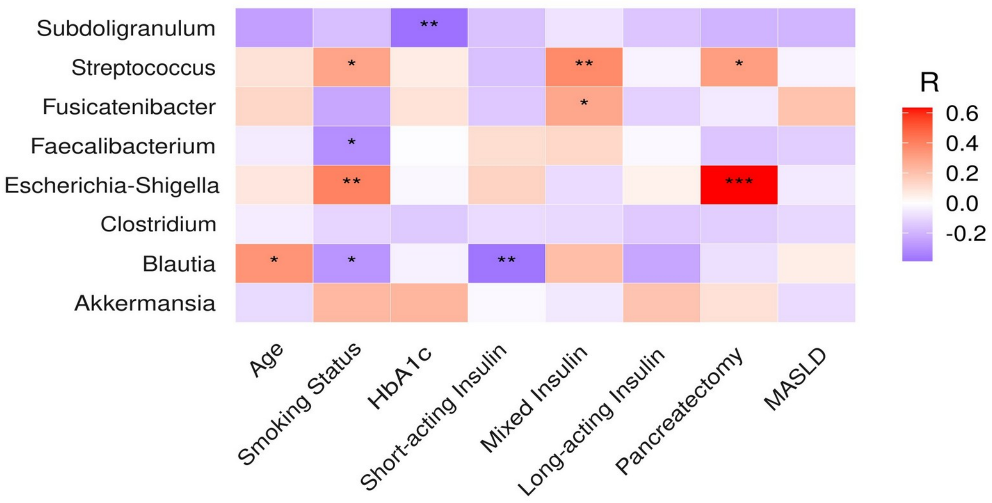

*p < 0.05 **p<0.01 ***p<0.001

Figure S8

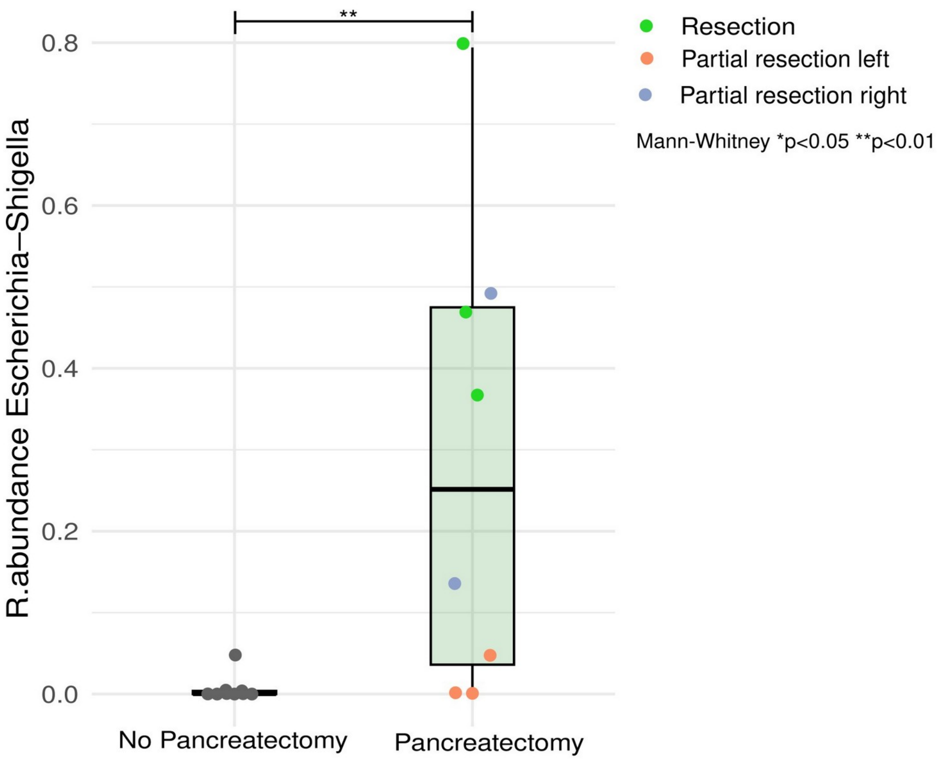
**
0.8
0.6
0.4
0.2
0.0
R.abundance Escherichia–Shigella
No Pancreatectomy
Pancreatectomy
Resection
Partial resection left
Partial resection right
Mann-Whitney *p<0.05 **p<0.01

Figure S9

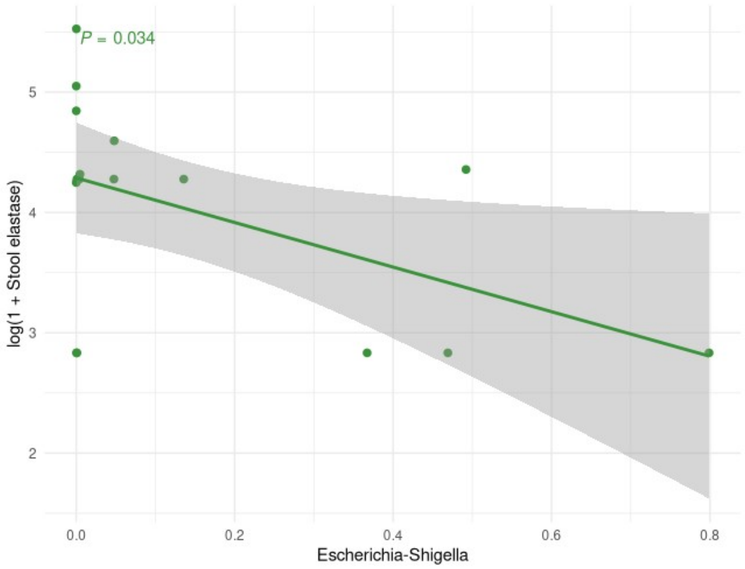
P = 0.034
log(1 + Stool elastase)
Escherichia-Shigella
5
4
3
2
0.0
0.2
0.4
0.6
0.8

Figure S10

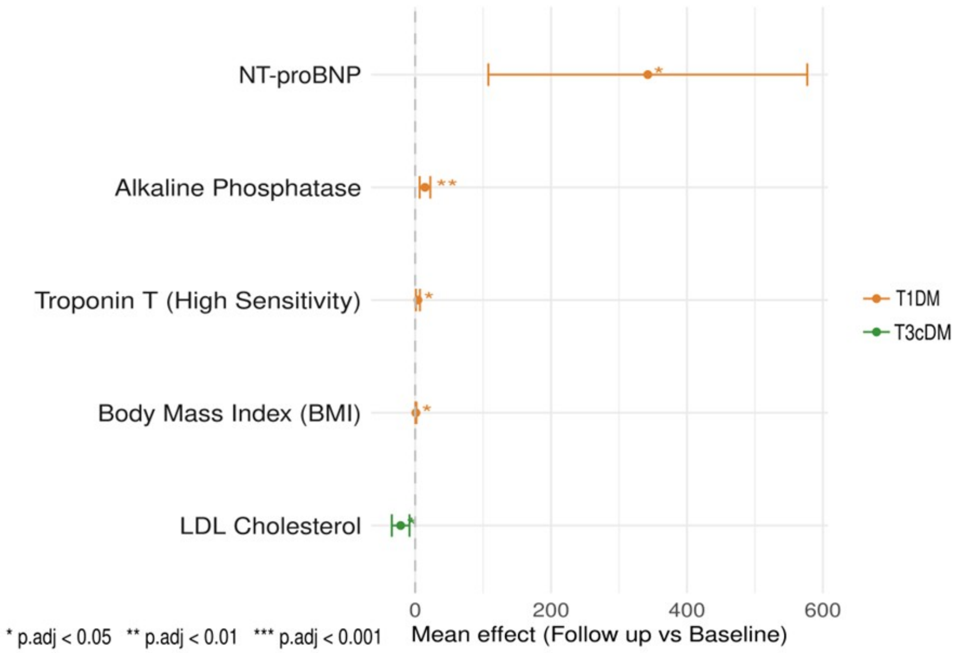
NT-proBNP
Alkaline Phosphatase
Troponin T (High Sensitivity)
Body Mass Index (BMI)
LDL Cholesterol
T1DM
T3cDM
0
200
400
600
Mean effect (Follow up vs Baseline)
* p.adj < 0.05 ** p.adj < 0.01 *** p.adj < 0.001

Figure S11

A

B

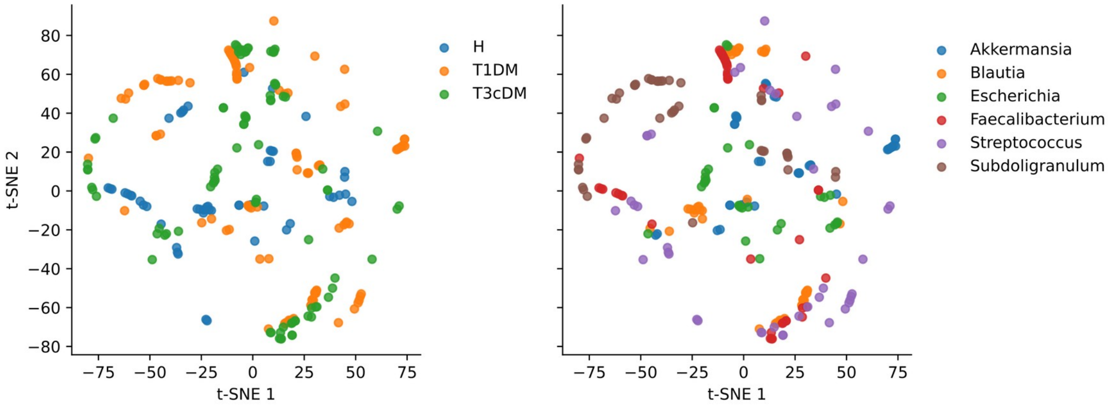

Figure S12

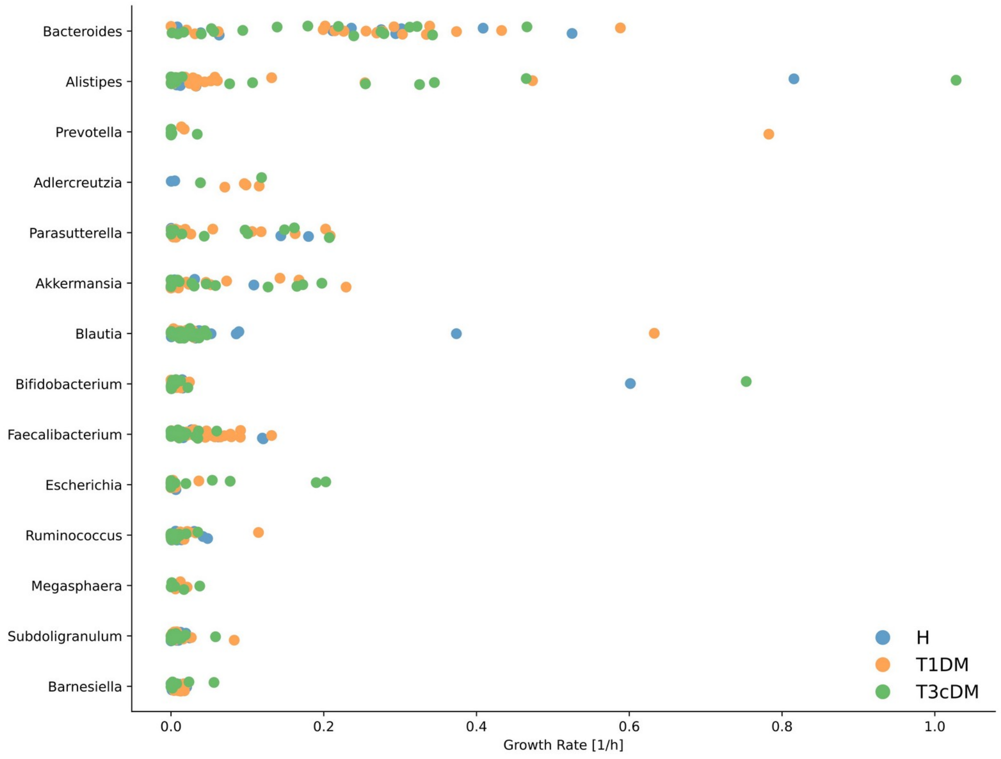
Bacteroides
Alistipes
Prevotella
Adlercreutzia
Parasutterella
Akkermansia
Blautia
Bifidobacterium
Faecalibacterium
Escherichia
Ruminococcus
Megasphaera
Subdoligranulum
Barnesiella
0.0
0.2
0.4
0.6
0.8
1.0
Growth Rate [1/h]
H
T1DM
T3cDM

Figure S13

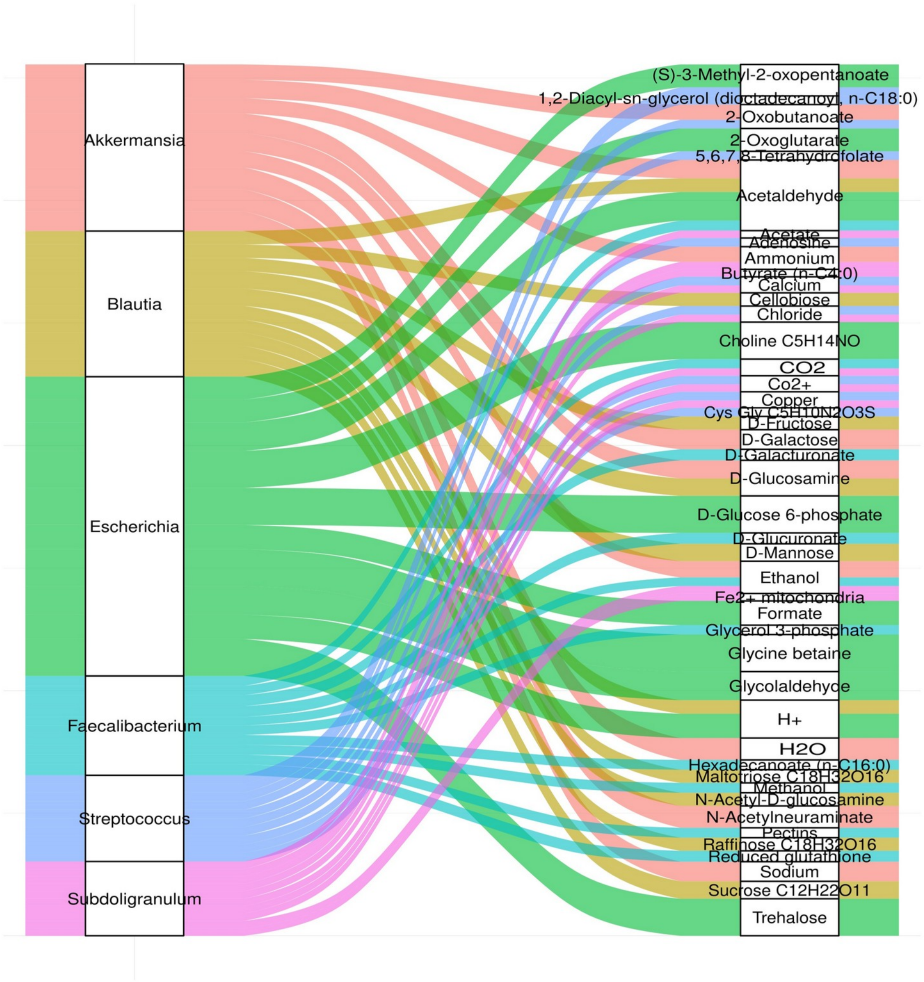
Akkermansia
Blautia
Escherichia
Faecalibacterium
Streptococcus
Subdoligranulum
(S)-3-Methyl-2-oxopentanoate
1,2-Diacyl-sn-glycerol (dioctadecanoyl, n-C18:0)
2-Oxobutanoate
2-Oxoglutarate
5,6,7,8-Tetrahydrofolate
Acetaldehyde
Acetate
Adenosine
Ammonium
Butyrate (n-C4:0)
Calcium
Cellobiose
Chloride
Choline C5H14NO
CO2
Co2+
Copper
Cys Gly C5H10N2O3S
D-Fructose
D-Galactose
D-Galacturonate
D-Glucosamine
D-Glucose 6-phosphate
D-Glucuronate
D-Mannose
Ethanol
Fe2+ mitochondria
Formate
Glycerol 3-phosphate
Glycine betaine
Glycolaldehyde
H+
H2O
Hexadecanoate (n-C16:0)
Maltotriose C18H32O16
Methanol
N-Acetyl-D-glucosamine
N-Acetylneuraminate
Pectins
Raffinose C18H32O16
Reduced glutathione
Sodium
Sucrose C12H22O11
Trehalose

Supplementary tables

Table 1. Patient characteristics

Clinical Parameter	T1DM [n = 21]	T3cDM [n = 17]	p-value
Sex [%]	F= 10 [48] M= 11 [52]	F= 7 [41] M= 10 [59]	0.388
Age	42 [+/- 13.3]	62 [+/- 10.1]	< **0.001**
mean [+/- SD]			
Weight			
mean [+/- SD]			
Baseline	71 [+/- 13.1]	71 [+/- 17.0]	0.905
Follow-up	76 [+/- 11.5; n = 21]	72 [+/- 11.5; n = 17]	0.400
Hight	173 [+/- 8.8]	171 [+/- 8.8]	0.542
mean [+/- SD]			
BMI			
mean [+/- SD]			
Baseline	24 [+/- 3.4]	24 [+/- 5.3]	0.896
Follow-up	25 [+/- 3.5]	24 [+/- 5.5]	0.296
Smoking [%]	5 [24]	12 [70]	**0.020**
Alcohol [%]	15 [71]	15 [88]	0.143

CAD			
Baseline [%]	6 [29]	10 [59]	0.222
Follow-up [%]	4 [19]	11 [65]	**0.014**
Arterial Hypertension			
Baseline [%]	7 [33]	14 [82]	**0.022**
Follow-up [%]	6 [29]	15 [88]	**<0.001**
MASLD			
Baseline [%]	2 [10]	9 [53]	**0.015**
Follow-up [%]	2 [10]	7 [41]	**0.061**
CA			
Baseline [%]	0	7 [41]	**0.003**
Follow-up [%]	0	7 [41]	**0.002**
Hyperlipidemia			
Baseline [%]	11 [52]	9 [52]	1.0
Follow-up [%]	16 [76]	9 [53]	0.156
Medication			
Insulin			
Baseline [%]	100 [n = 21]	100 [n = 17]	
Follow-up [%]	100 [n = 21]	100 [n = 17]	

Diuretics			
Baseline [%]	0	5 [29]	**0.019**
Follow-up [%]	0	2 [12]	0.166
Antihypertensive agents			
Baseline [%]	7 [33]	15 [88]	**0.010**
Follow-up [%]	6 [28]	14 [82]	**<0.001**
Lipid-lowering agents			
Baseline [%]	11 [53]	9 [53]	1.0
Follow-up [%]	16 [76]	9 [53]	0.246

T1DM, Type 1 diabetes mellitus; T3cDM, pancreatogenic diabetes mellitus; n, Number; BMI, body mass index; CAD, coronary Artery Disease; MASLD, metabolic dysfunction-associated steatotic liver disease, CA, ACE-Inhibitors, angiotensin-converting-enzyme inhibitors; clinical follow up parameter: 1 patient in the T3cDM group is missing.

Table 2. Laboratory Parameters

Laboratory value [mean; +/- SD]	T1DM	T3cDM	p-value
Leukocytes [G/l]			
Baseline	6.8 [+/- 2.6; n = 21]	7.7 [+/- 2.4; n = 17]	0.215
Follow-up	6.6 [+/- 2.3; n = 21]	7.7 [+/- 2.9; n = 17]	0.226
Hemoglobin [g/l]			
Baseline	144 [+/- 12.0; n = 21]	140 [+/- 12.7; n = 17]	0.176
Follow-up	140 [+/- 16.4; n = 21]	141 [+/- 11.3; n = 17]	0.424
Hematocrit [l/l]			
Baseline	0.41 [+/- 0.03; n = 21]	0.41 [+/- 0.04; n = 17]	0.394
Follow-up	0.41 [+/- 0.04; n = 21]	0.41 [+/- 0.04; n = 17]	0.812
Thrombocytes [G/l]			
Baseline	218 [+/- 72; n = 21]	257 [+/- 94; n = 17]	0.189
Follow-up	241 [+/- 87.4; n = 21]	260 [+/- 75.7; n = 17]	0.291
Urea [mg/dl]			
Baseline	32.6 [+/- 10.1; n = 21]	34.5 [+/- 10.8; n = 17]	0.406
Follow-up	36.3 [+/- 16.3; n = 21]	39.1 [+/- 14.9; n = 17]	0.238

Creatinine [mg/dl]			
Baseline	0.97 [+/- 0.31; n = 21]	0.88 [+/- 0.25; n = 17]	0.054
Follow-up	0.98 [+/- 035; n = 21]	0.80 [+/- 0.29; n = 17]	**0.007**
GFR [ml/min/1,73m^2]			
Baseline	58 [+/- 6.8; n = 21]	58 [+/- 4.5; n = 17]	0.223
Follow-up	58 [+/- 7.1; n = 21]	59 [+/- 3.5; n = 17]	0.885
Total Bilirubin [mg/dl]			
Baseline	0.58 [+/- 0.23; n = 21]	0.67 [+/- 0.27; n = 17]	0.189
Follow-up	0.64 [+/- 0.34; n = 21]	0.69 [+/- 0.48; n = 17]	0.686
Sodium [mmol/l]			
Baseline	139 [+/- 3.5; n = 21]	137 [+/- 6.6; n = 17]	0.263
Follow-up	138 [+/- 2.4; n = 21]	138 [+/- 4.2; n = 17]	0.884
AST [U/l]			
Baseline	23 [+/- 9.0; n = 21]	33 [+/- 23.2; n = 17]	**0.045**
Follow-up	33 [+/- 24.7; n = 21]	69 [+/- 87.0; n = 17]	**0.016**
ALT [U/l]			
Baseline	21 [+/- 11.2; n = 21]	31 [+/- 30.3; n = 17]	0.111
Follow-up	30 [+/- 26.0; n = 21]	62 [+/- 92.0; n = 17]	0.250
GGT [U/l]			
Baseline	39 [+/- 48.7; n = 21]	62 [+/- 98.2; n = 17]	**0.041**

Follow-up	67 [+/- 136.0; n = 21]	199 [+/- 621.7; n = 17]	0.100
ALP [U/l]			
Baseline	68 [+/- 23.8; n = 21]	89 [+/- 38.5; n = 17]	**0.030**
Follow-up	84 [+/- 34.0; n = 21]	155 [+/- 233.6; n = 17]	**0.014**
LDH [U/l]			
Baseline	194 [+/- 47.2; n = 21]	217 [+/- 63.8; n = 17]	0.142
Follow-up	214 [+/- 78.6; n = 21]	259 [+/- 74.1; n = 17]	0.075
CRP [mg/dl]			
Baseline	0.42 [+/- 0.60; n = 21]	0.78 [+/- 1.43; n = 23]	0.526
Follow-up	0.47 [+/- 0.71; n = 21]	0.51 [+/- 0.82; n = 23]	0.827
INR			
Baseline	1.0 [+/- 0.066; n = 21]	1.0 [+/- 0.077; n = 17]	0.571
Follow-up	1.0 [+/- 0.068; n = 21]	1.0 [+/- 0.062; n = 17]	0.665
PTT [s]			
Baseline	31 [+/- 6.9; n = 21]	30 [+/- 3.9; n = 17]	0.984
Follow-up	34 [+/- 7.7; n = 21]	32 [+/- 5.6; n = 17]	0.311
Fibrinogen [mg/dl]			
Baseline	284 [+/- 85.1; n = 21]	351 [+/- 108.2; n = 17]	**0.028**
Follow-up	318 [+/- 80.4; n = 21]	247 [+/- 61.8; n = 17]	0.188
Albumin [mg/dl]			
Baseline	3968 [+/- 298.9; n = 21]	3907 [+/- 499.2; n = 17]	0.880

Follow-up	4025 [+/- 283.1; n = 21]	3999 [+/- 324.7; n = 17]	0.828
Glucose [mg/dl]			
Baseline	165 [+/- 81.2; n = 21]	168 [+/- 108.7; n = 17]	0.952
Follow-up	152 [+/- 59.8; n = 21]	155 [+/- 72.4; n = 17]	0.869
HbA1c [%]			
Baseline	7.2 [+/- 1.1; n = 21]	8.4 [+/- 2.2; n = 17]	**0.035**
Follow-up	7.2 [+/- 0.9; n = 21]	8.4 [+/- 1.1; n = 17]	**< 0.001**
Serum TC [mg/dl]			
Baseline	170 [+/- 34.9; n = 21]	173 [+/- 26.2; n = 17]	0.785
Follow-up	162 [+/- 38.0; n = 21]	156 [+/- 43.6; n = 17]	0.780
Triglycerides [mg/dl]			
Baseline	87 [+/- 44.8; n = 21]	115 [+/- 75.3; n = 17]	0.130
Follow-up	71 [+/- 22.2; n = 21]	88 [+/- 30.2; n = 17]	**0.038**
HDL-Cholesterol [mg/dl]			
Baseline	64 [+/- 18.1; n = 21]	59 [+/- 16.1; n = 17]	0.289
Follow-up	68 [+/- 14.6; n = 21]	65 [+/- 18.4; n = 17]	0.537
LDL-Cholesterol [mg/dl]			
Baseline	100 [+/- 33.2; n = 21]	98 [+/- 24.8; n = 17]	0.853
Follow-up	86 [+/- 37.8; n = 21]	75 [+/- 29.3; n = 17]	0.306

Iron [µmol/l]			
Baseline	18 [+/- 7.0; n = 21]	15 [+/- 6.4; n = 17]	**0.050**
Follow-up	20 [+/- 7.7; n = 21]	18 [+/- 10.0; n = 17]	**0.017**
Serum Ferritin [µg/l]			
Baseline	118 [+/- 93.9; n = 21]	88 [+/- 85.5; n = 17]	0.179
Follow-up	193 [+/- 268.0; n = 21]	162 [+/- 207.0; n = 17]	0.788
Serum Transferrin [mg/dl]			
Baseline	220 [+/- 34.4; n = 21]	265 [+/- 51.5; n = 17]	**<0.001**
Follow-up	243 [+/- 77.8; n = 21]	263 [+/- 72.5; n = 17]	**0.027**
TSAT [%]			
Baseline	34 [+/- 13.6; n = 21]	25 [+/- 14.4; n = 17]	**0.035**
Follow-up	37 [+/- 19.5; n = 21]	33 [+/- 19.0; n = 17]	0.362
cTNT [ng/l]			
Baseline	6.1 [+/- 1.6; n = 21]	11.6 [+/- 9.5; n = 17]	**0.020**
Follow-up	10 [+/- 6.1; n = 21]	17 [+/- 14.6; n = 17]	**0.083**
NT-proBNP [ng/l]			
Baseline	158 [+/- 139.4; n = 21]	294 [+/- 332.2; n = 17]	**0.058**
Follow-up	464 [+/- 473.5; n = 21]	838 [+/- 943.4; n = 17]	0.171

T1DM, Type 1 diabetes mellitus; T3cDM, pancreatogenic diabetes mellitus; n, Number; SD, standard deviation; GFR, glomerular filtration rate; AST, aspartate aminotransferase; ALT, alanine aminotransferase; GGT, gamma-glutamyl transferase; ALP, alkaline phosphatase; LDH, lactate-dehydrogenase; CRP, C-reactive Protein; INR, international normalized ratio; PTT, partial thromboplastin time; HbA1c, hemoglobin A1c; Serum TC, total cholesterol; cTNT, cardiac troponin T; NT-proBNP, N-terminal pro–B-type natriuretic peptide; TSAT, transferrin saturation

Table 3. Healthy Control (n=10)

Clinical Parameter	
Sex [%]	F= 5 [50] M= 5 [50]
Age mean [+/- SD]	56 [+/- 15.6]
Weight mean [+/- SD]	
Baseline	69 [+/- 11.6]
Follow-up	70 [+/- 8.8]
Hight mean [+/- SD]	171 [+/- 8]
BMI mean [+/- SD]	
Baseline	24 [+/- 1.4]
Follow-up	24 [+/- 0.9]
Smoking	none
Alcohol consumption	none
CAD	
Baseline	none
Follow-up	none

Arterial Hypertension	
Baseline	none
Follow-up	none
MASLD	
Baseline	none
Follow-up	none
CA	
Baseline	none
Follow-up	none
Hyperlipidemia	
Baseline	none
Follow-up	none
Laboratory Parameter [mean; +/- SD]	
Leukocytes [G/l]	
Baseline	6.2 [+/- 1.4]
Follow-up	6.7 [+/- 0.9]
Hemoglobin [g/l]	
Baseline	142 [+/- 8.0]
Follow-up	141.3 [+/- 4.4]
Hematocrit [l/l]	

Baseline	0.42 [+/- 0.03]
Follow-up	0.43 [+/- 0.03]
Thrombocytes [G/l]	
Baseline	267.5 [+/- 64.0]
Follow-up	335.0 [+/- 34.6]
Urea [mg/dl]	
Baseline	62.4 [+/- 22.3]
Follow-up	66.7 [+/- 11.6]
Creatinine [mg/dl]	
Baseline	0.7 [+/- 0.2]
Follow-up	0.7 [+/- 0.2]
GFR [ml/min/1,73m^2]	
Baseline	60 [+/- 0]
Follow-up	60 [+/- 0]
Total Bilirubin [mg/dl]	
Baseline	0.6 [+/- 0.2]
Follow-up	0.6 [+/- 0.2]
Sodium [mmol/l]	
Baseline	136.8 [+/- 2.8]
Follow-up	137.9 [+/- 2.6]

AST [U/l]	
Baseline	23.0 [+/- 4.4]
Follow-up	24.2 [+/- 5.8]
ALT [U/l]	
Baseline	21.3 [+/- 7.1]
Follow-up	23.0 [+/- 4.3]
GGT [U/l]	
Baseline	72.7 [+/- 24.0]
Follow-up	28.5 [+/- 3.9]
ALP [U/l]	
Baseline	199.5 [+/- 24.7]
Follow-up	92.8 [+/- 23.0]
LDH [U/l]	
Baseline	99.0 [+/- 1.9]
Follow-up	195.1 [+/- 28.6]
CRP [mg/dl]	
Baseline	0.14 [+/- 0.13]
Follow-up	0.19 [+/- 0.18]
INR	
Baseline	1.0 [+/- 0.06]

Follow-up	1.0 [+/- 0.08]
PTT [s]	
Baseline	30 [+/- 2.4]
Follow-up	32.5 [+/- 3.8]
Fibrinogen [mg/dl]	
Baseline	225.1 [+/- 35.4]
Follow-up	271.1 [+/- 33.1]
Albumin [mg/dl]	
Baseline	4112 [+/- 78.4]
Follow-up	4109 [+/- 110.0]
Glucose [mg/dl]	
Baseline	85.5 [+/- 11.5]
Follow-up	88.7 [+/- 5.3]
HbA1c [%]	
Baseline	4.5 [+/- 0.5]
Follow-up	4.6 [+/- 0.6]
Serum TC [mg/dl]	
Baseline	40.7 [+/- 2.4]
Follow-up	41.5 [+/- 2.8]
Triglycerides [mg/dl]	

Baseline	109.9 [+/- 7.7]
Follow-up	108.7 [+/- 7.5]
LDL-Cholesterol [mg/dl]	
Baseline	85.6 [+/- 13.5]
Follow-up	94.3 [+/- 7.5]
Iron [µmol/l]	
Baseline	24.2 [+/- 3.9]
Follow-up	25.5 [+/- 2.2]
Serum Ferritin [µg/l]	
Baseline	145.5 [+/- 53.5]
Follow-up	120.9 [+/- 25.2]
Serum Transferrin [mg/dl]	
Baseline	223.0 [+/- 28.8]
Follow-up	232.7 [+/- 29.9]
TSAT [%]	
Baseline	40.1 [+/- 7.6]
Follow-up	39.9 [+/-5.2]
cTNT [ng/l]	
Baseline	0.5 [+/- 0]

Follow-up	0.5 [+/- 0]
NT-proBNP [ng/l]	
Baseline	50 [+/- 0]
Follow-up	50 [+/- 0]

SD, standard deviation; GFR, glomerular filtration rate; AST, aspartate aminotransferase; ALT, alanine aminotransferase; GGT, gamma-glutamyl transferase; ALP, alkaline phosphatase; LDH, lactate-dehydrogenase; CRP, C-reactive Protein; INR, international normalized ratio; PTT, partial thromboplastin time; HbA1c, hemoglobin A1c; Serum TC, total cholesterol; cTNT, cardiac troponin T; NT-proBNP, n-terminal pro–B-type natriuretic peptide; TSAT, transferrin saturation; BMI, body mass index; CAD, coronary artery disease; MASLD, metabolic dysfunction-associated steatotic liver disease, CA, carinoma

Table 4. Power table for sample size and statistical power estimation

N per group	Statistical power
6	0.356
8	0.488
10	0.706
12	0.878
14	0.974
16	1.0
17	1.0

Additional methods

1. Sample and data collection

All samples were collected during routine clinical visits from patients, as well as from a healthy control who was referred to our department for a screening colonoscopy. An antibiotics or probiotics free period of 6 to 12 weeks prior to the stool sample was defined as a primary eligibility requirement. The patient's regular medication, comorbidities, smoking habits, daily alcohol consumption and additional clinical parameters (e.g. body mass index (BMI)) were obtained by the patients report or from the electronic health record and within an excel dataset on two different time points. Information on patients' sex assigned at birth was extracted from the electronic health records; we did not assess other gender identities. All participants signed an informed consent form on the day of their routine appointment.

1.1 Inclusion and exclusion criteria

Adults ≥18 years with a confirmed diagnosis of type 1 diabetes mellitus or type 3c diabetes who were treated at the outpatient clinic of the Department of Internal Medicine I (Gastroenterology, Hepatology, Endocrinology and Metabolism) at the Medical University of Innsbruck during routine clinical follow-up visits were eligible for inclusion. All participants were fully insulin-dependent and provided written informed consent prior to study participation.

Patients were excluded if they were <18 years of age, did not provide written informed consent, were not fully insulin-dependent, or had other forms of diabetes mellitus, including type 2 diabetes mellitus, gestational diabetes, monogenic diabetes (e.g., maturity-onset diabetes of the young [MODY]), diabetes due to other specific causes such as endocrinopathies (e.g., Cushing syndrome or acromegaly), drug- or chemical-induced diabetes (e.g., glucocorticoids), genetic defects of insulin action, or other rare specific types of diabetes, according to established classification guidelines[1].

1.2 Assessment of exocrine pancreatic insufficiency symptoms

Symptoms suggestive of Exocrine Pancreatic Insufficiency (EPI) were assessed during routine clinical evaluation using a standardized symptom questionnaire. Patients were specifically asked about gastrointestinal symptoms associated with pancreatic exocrine dysfunction, including steatorrhea (fatty or oily stools), chronic diarrhea, abdominal bloating, excessive flatulence, abdominal discomfort, and unintentional weight loss. In addition, clinical indicators of malabsorption such as fat-soluble vitamin deficiency and nutritional impairment were recorded when available in the medical records. Symptom assessment was performed as part of routine outpatient monitoring and documented in the study database for subsequent analysis [2]. In addition, stool elastase was measured to objectively evaluate pancreatic exocrine function. A cutoff of <200 µg/g stool was used to define EPI, in accordance with established guidelines. Patients with stool elastase values below this threshold were classified as having exocrine pancreatic insufficiency [3].

1.3 Blood samples

Peripheral whole blood samples collected from patients/healthy controls during routine clinical monitoring. Serum was prepared within 2 hours after blood collection by centrifugation at 1200 g for 15 minutes at 20 °C using a Universal 320/320R centrifuge (Hettich, Tuttlingen, Germany). The resulting serum was stored at −80 °C until further analysis.

1.4 Fecal samples

Fecal samples were collected from all patients/healthy controls included in the study. Participants collected approximately 50 mg of stool and placed it into three sterile tubes that had been provided to them in advance. Samples were immediately stored at −20 °C (fresh frozen), and the cold chain was maintained at −20 °C using transportable cooling bags during transport to the laboratory. Upon arrival, the samples were transferred to −80

°C for long-term storage. During routine clinical monitoring, participants also completed a questionnaire including the Bristol Stool Scale.

2. Fecal DNA extraction and sequencing

Stool DNA was extracted following protocol Q [4] , using mechanical, thermal, chemical, and enzymatic lysis together with bead-beating, heat treatments, and centrifugation. DNA was purified by ammonium acetate precipitation, ethanol washes, and column-based cleanup using the QIAamp Fast DNA Stool Mini Kit (Qiagen). Quantification was performed using a NanoDrop 1000 spectrophotometer (Thermo Fisher Scientific), and stored at −80°C until sequencing. For 16S rRNA amplicon sequencing, DNA was diluted to 100 ng/10 µl and processed by IMGM Laboratories (Germany) using primers F_NXT_Bakt_341F and R_NXT_Bakt_805R [5] targeting V3–V4 (464 bp). Amplicon quality was assessed by 2% agarose gel electrophoresis, purified (AMPure XP beads), quantified (Qubit dsDNA HS), pooled equimolarly (SequalPrep Normalization Plate Kit), and assessed on an Agilent Bioanalyzer. Libraries were denatured with NaOH and sequenced on an Illumina MiSeq (v2 kit, 500 cycles, 10% PhiX), generating paired-end 2 × 250 bp reads by sequencing-by-synthesis (SBS).

3. 16S rRNA gene sequencing data analysis

Raw paired-end 16S rRNA sequencing data from 21 individuals with T1DM, 17 with T3cDM, and 10 healthy controls (H) were processed with nf-core/ampliseq pipeline v2.12.0 implemented in Nextflow. This pipeline wraps all analysis steps and software and is publicly available at https://nf-co.re/ampliseq [6]. Primer trimming was performed using cutadapt v.4.6 [7]. The primer pair was: forward =5′–CCTACGGGNGGCWGCAG–3′ and reverse =5′–GACTACHVGGGTATCTAATCC–3′. Quality control was performed using FastQC v0.12.1 [8]. Reads were filtered for quality and length prior to downstream analysis. Denoising, inference of amplicon sequence variants (ASVs) and taxonomical classification were performed with DADA2 v1.30.0 [9]. The filtered sequences were deduplicated and aligned against the V3-V4 region of

the SILVA v138.1 reference small subunit rRNA gene alignment database [10]. Using QIIME2 v2023.7.0 unwanted taxa were excluded, absolute and relative feature/taxa count tables were computed together with alpha and beta diversity indices [11]. The pipeline also created phyloseq R v4.3.0 objects that were used for downstream analysis [12,13].

With the available sample sizes (T1DM n = 21; T3cDM n = 17), we estimated the statistical power using a Monte Carlo resampling approach with 500 simulations. In each simulation, Bray–Curtis dissimilarities were calculated and group differences in microbial community composition were tested using PERMANOVA. Statistical power was defined as the proportion of simulations producing a statistically significant result ($p < 0.05$). Based on the variability observed in our dataset, the estimated statistical power exceeded 90%. Additional details are provided in Supplementary Table 4.

For the downstream analysis taxa were aggregated at the genus level filtered (mean rel. abundance >3%, prevalence >5%) and normalized. Statistical significance was assessed using Mann-Whitney-Test for α-diversity and PERMANOVA for β-diversity. Linear Discriminant Analysis Effect Size (LefSe) was used to compare the relative abundance of different taxa and identify those most likely to distinguish between diseases in the pairwise comparisons: T1DM vs T3cDM, T1DM vs healthy controls (H), and T3cDM vs healthy controls. LefSe was also used to study the sex comparison (male n=28 vs female n=21) within each disease group separately. Pairwise comparisons using Mann-Whitney-Test were performed on top genera relative abundance identified from logistic models and visualized as boxplots. Pearson correlation analyses were performed between relative abundances of top genera identified from logistic models and selected clinical variables relevant to T1DM and T3cDM. Correlations were corrected for multiple testing using the Benjamini–Hochberg method (*p.adj < 0.05, **p.adj < 0.01, ***p.adj < 0.001).

Linear regression was fitted and p value calculated to evaluate the relationship between Escherichia-Shigella relative abundance and the binary clinical variable "Pancreatectomy". This analysis is complementary to a group-wise comparison across Pancreatectomy resection types. Among the T3cDM patients, 8 underwent pancreatectomy (3 total resection, 3 partial resection left, and 2 partial resection right),

whereas 9 patients did not underwent pancreatectomy. This was visualized using boxplots; p-values were omitted due to limited sample size.

Clinical and laboratory variables collected at baseline and follow-up previously anonymized were analyzed using generalized linear mixed-effects models (GLMM) for binary outcomes [14]. All patients from T1DM (n=21) and T3cDM (n=17) were included in this analysis. Fixed effects included time, age, sex, and clinical group. Random intercepts were modeled per patient. Effect estimates and 95% confidence intervals were extracted and significance was adjusted via Benjamini–Hochberg method. Results were visualized as forest plots.

References

1. American Diabetes Association. Classification and Diagnosis of Diabetes: *Standards of Care in Diabetes*. Diabetes Care. 2024.

2. United European Gastroenterology HaPanEU/UEG Working Group. *United European Gastroenterology evidence-based guidelines for the diagnosis and therapy of chronic pancreatitis.* United European Gastroenterology Journal. 2017.

3. Lankisch, P. G., et al. *Exocrine pancreatic insufficiency: Diagnostic evaluation and clinical management.* World Journal of Gastroenterology. 2015;21(42):12043–12054.

4. Costea PI, Zeller G, Sunagawa S, Pelletier E, Alberti A, Levenez F, et al. Towards standards for human fecal sample processing in metagenomic studies. *Nat Biotechnol.* 2017;35(11):1069–1076.
5. Klindworth A, Pruesse E, Schweer T, Peplies J, Quast C, Horn M, et al. Evaluation of general 16S ribosomal RNA gene PCR primers for classical and next-generation sequencing-based diversity studies. *Nucleic Acids Res.* 2013;41(1):e1.
6. Ewels PA, Peltzer A, Fillinger S, Patel H, Alneberg J, Wilm A, et al. The nf-core framework for community-curated bioinformatics pipelines. *Nat Biotechnol.* 2020;38:276–278.
7. Martin M. Cutadapt removes adapter sequences from high-throughput sequencing reads. *EMBnet J.* 2011;17(1):10–12.
8. Babraham BF. FastQC: a quality control tool for high throughput sequence data. Babraham Institute. 2011.
9. Callahan BJ, McMurdie PJ, Rosen MJ, Han AW, Johnson AJA, Holmes SP. DADA2: high-resolution sample inference from Illumina amplicon data. *Nat Methods.* 2016;13(7):581–583. doi:10.1038/nmeth.3869.
10. Quast C, Pruesse E, Yilmaz P, Gerken J, Schweer T, Yarza P, et al. The SILVA ribosomal RNA gene database project: improved data processing and web-based tools. *Nucleic Acids Res.* 2013;41(Database issue):D590–D596.

11. Bolyen E, Rideout JR, Dillon MR, Bokulich NA, Abnet CC, Al-Ghalith GA, et al. Reproducible, interactive, scalable and extensible microbiome data science using QIIME 2. *Nat Biotechnol.* 2019;37:852–857.
12. McMurdie PJ, Holmes S. phyloseq: an R package for reproducible interactive analysis and graphics of microbiome census data. *PLoS One*. 2013;8(4):e61217.
13. R Core Team. R: a language and environment for statistical computing. Vienna: R Foundation for Statistical Computing; 2014.
14. Bates D, Mächler M, Bolker BM, Walker SC. Fitting linear mixed-effects models using lme4. *J Stat Softw*. 2015;67(1):1–48.
